# MiR-7 in neurological disorders: beyond metabolic dysregulation

**DOI:** 10.3389/fnins.2026.1884638

**Published:** 2026-07-30

**Authors:** Cristina Puigdueta, Virginia Pardo-Marqués, Marta Torrecilla-Parra, Mario Fernández-de Frutos, José L. López-Aceituno, Carmen Zamora, Yaiza López-Manchedo, Rebeca Busto, Cristina M. Ramírez

**Affiliations:** 1 Posttranscriptional Regulation of Metabolic Diseases Laboratory, Department of Precision Nutrition and Aging, IMDEA Nutrition Research Institute, Madrid, Spain; 2 IMDEA Nutrition Research Institute, Madrid, Spain; 3 Department of Biochemistry-Research, Hospital Universitario Ramón y Cajal, IRYCIS, Madrid, Spain

**Keywords:** brain diseases, metabolism, miR-7, neurodegeneration, physiological function, posttranscriptional regulation, tumor suppressor

## Abstract

Brain health depends on the fine coordination of metabolic and neuronal pathways, and disturbances in this balance are recognized as major drivers of neurodegenerative, neurological, and neuropsychiatric disorders. Increasing evidence indicates that microRNAs (miRNAs), as key post-transcriptional regulators of gene expression, play important roles in the molecular networks underlying neurological diseases by modulating pathways involved in energy metabolism, inflammation, oxidative stress, and neuronal survival. Among them, miR-7 has emerged as a particularly relevant regulator due to its high conservation, abundant expression in the central nervous system (CNS), and pleiotropic actions impacting both metabolic and neurodegenerative processes. This review focuses on current knowledge regarding miR-7 as a molecular node linking metabolic imbalance to neurological dysfunction, highlighting its regulatory targets and potential translational relevance in neurometabolic disorders.

## 1 Introduction

Metabolic homeostasis is essential for brain function, as the CNS has exceptionally high energy requirements relative to its mass and depends on a continuous supply of nutrients to sustain neuronal activity. Because cerebral glycogen reserves are minimal, neurons rely largely on continuous glucose availability together with efficient mitochondrial oxidative metabolism to meet their energetic demands (Sonnay et al., 2017). Consequently, even subtle disturbances in substrate utilization, mitochondrial function, or cellular energy sensing may compromise neuronal activity and progressively alter brain homeostasis. Although glucose represents the principal immediate energy substrate for the brain, lipid metabolism is equally important for maintaining neural integrity. Lipids fulfill indispensable structural and signaling functions, as major components of neuronal and glial membranes, synaptic vesicles, and myelin, while also serving as precursors for multiple bioactive mediators (Tracey et al., 2018). Cholesterol, phospholipids, fatty acids, and sphingolipids are particularly abundant in the nervous system, where their tightly regulated composition determines membrane fluidity, receptor organization, vesicular trafficking, and signal transduction. Therefore, disturbances in lipid handling may affect not only membrane architecture but also synaptic communication, inflammatory responses, and neuronal plasticity (Goedeke and Fernández-Hernando, 2012).

Accumulating evidence indicates that metabolic dysfunction is not merely a secondary consequence of neurological disease but often represents an early and integral component of its pathogenesis. Alzheimer’s disease (AD) provides one of the clearest examples, as reduced cerebral glucose utilization is among the earliest detectable abnormalities and precedes neurodegeneration (Christodoulou et al., 2026). In parallel, alterations in lipid homeostasis, including changes in cholesterol composition and sphingolipid balance (Rudajev and Novotny, 2022), contribute to pathological amyloid processing and synaptic dysfunction, two hallmarks of disease progression. Similar metabolic perturbations can extend to other neurodegenerative disorders such as Parkinson’s disease (PD), Amyotrophic lateral sclerosis (ALS), and Huntington’s disease (HD), all of which display varying degrees of mitochondrial dysfunction, impaired neuronal glucose handling, oxidative imbalance or lipid metabolism. This metabolic dimension also encompasses neuropsychiatric disorders such as Schizophrenia (SCZ), which have been associated with altered membrane lipid composition and impaired bioenergetics (Dong-Chen et al., 2023; Nelson and Trotti, 2022; Singh and Agrawal, 2022; Roosterman and Cottrell, 2021), or even certain type of brain tumors (Pavlova et al., 2022). At the cellular level, other processes further amplify neurometabolic dysfunction such as protein control pathways including proteostasis and autophagy (Pavlova et al., 2022).

In addition to the intrinsic metabolic defect found in these neurological disorders, systemic metabolic dysfunction also impacts brain function. This interconnection is mediated by metabolic signals such as insulin, leptin, and ghrelin actively modulate neuronal survival, stress responses, substrate utilization, and synaptic adaptation, underscoring the bidirectional communication between peripheral energy balance and brain function. The brain systemic metabolism interplay becomes particularly relevant in conditions such as obesity, insulin resistance, and Type 2 Diabetes (T2D), which contribute to neurological vulnerability and the development of neurodegenerative disorders (Heni, 2024; Kacem et al., 2025).

**Figure 1:**
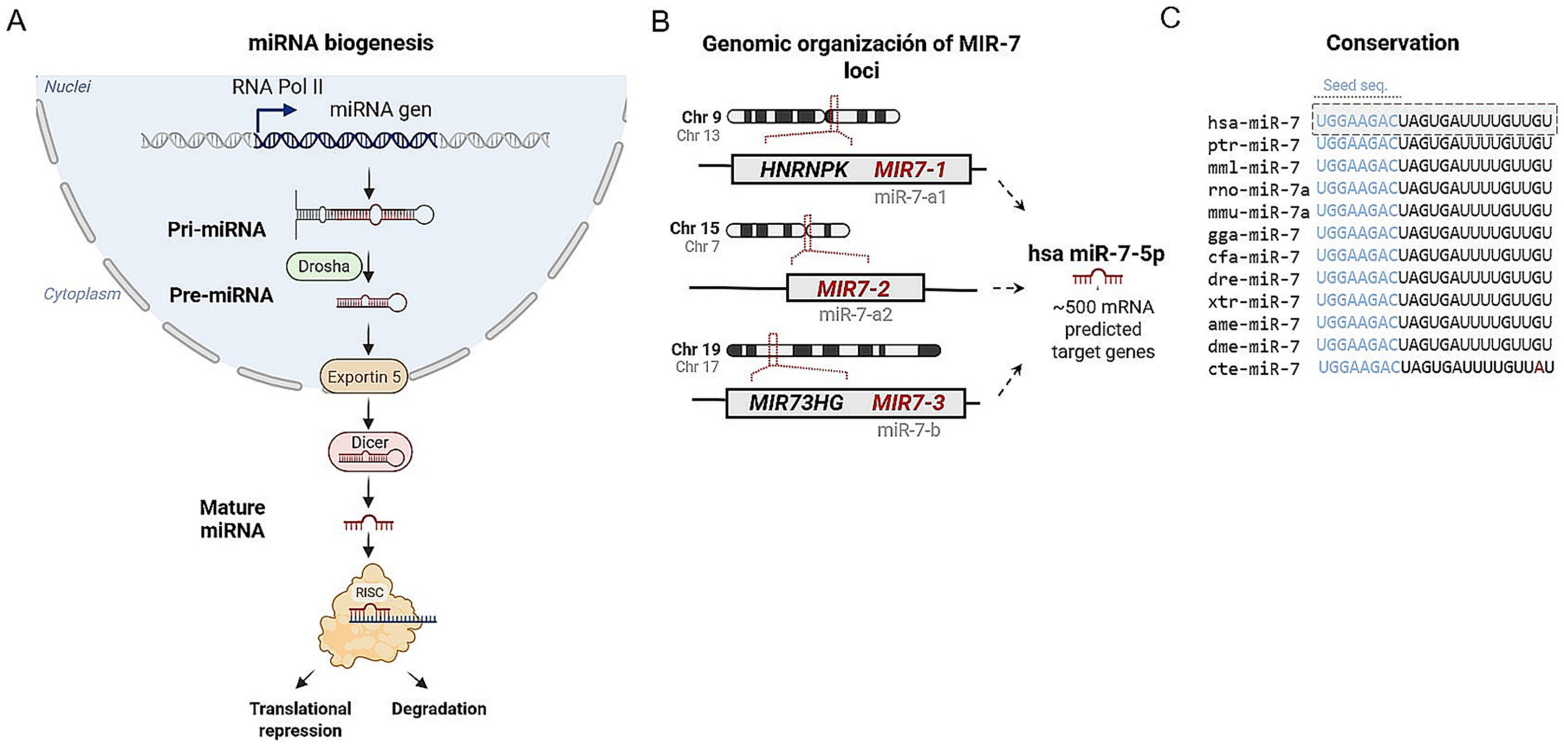
Biogenesis, genomic organization and evolutionary conservation of miR-7. **(A)** Canonical miRNA biogenesis pathway. Most miRNA genes are transcribed by RNA polymerase II (RNA Pol II) as primary transcripts (pri-miRNAs), which are sequentially processed by the RNase III-endonuclease Drosha to generate precursor miRNAs (pre-miRNAs). Following Exportin-5-mediated nuclear export, Dicer cleavage produces the mature miRNA duplex, from which the guide strand is incorporated into the RNA-induced silencing complex (RISC) to mediate translational repression and/or mRNA degradation of target transcripts. MiR-7 follows this canonical biogenesis pathway. **(B)** Genomic organization of the three human MIR7 loci. MIR7-1 is located on chromosome 9 within an intron of the HNRNPK gene, MIR7-2 is located on chromosome 15 as an intergenic locus, and MIR7-3 is embedded within the MIR7-3HG host gene on chromosome 19. The corresponding murine genomic organization, including chromosomal locations and miR-7 orthologues (miR-7a1, miR-7a2, and miR-7b), is shown in gray. Despite their distinct genomic origins, all three human loci generate the identical mature hsa-miR-7-5p sequence, which is predicted to regulate approximately 500 target genes. **(C)** Evolutionary conservation of the mature miR-7 sequence across representative vertebrate and invertebrate species. The seed region (highlighted in blue) is highly conserved, underscoring the remarkable evolutionary conservation of miR-7 and its biological functions. Created with BioRender.com.

The metabolic impairment associated with these neurological diseases has commonly been linked to defects in gene expression, which compromise the tight control of the complex reactions and processes governing cellular homeostasis. Besides classic transcriptional mechanisms, miRNAs function as central modulators of gene expression at the posttranscriptional level (Cao et al., 2016). These small evolutionarily conserved non-coding RNAs, typically 18–25 nucleotides in length, are generated through a multistep biogenesis process involving Drosha- and Dicer-mediated processing (Figure 1A) (Ambros, 2004). They predominantly act through complementary binding to sequences within the 3′ untranslated region (3′ UTR) of target messenger RNAs (mRNAs), resulting in translational repression, transcript destabilization, or both (Ambros, 2004). Because of their ability to regulate broad gene networks, rather than individual targets, these molecules control multiple cellular functions, including insulin signaling, glucose transport, mitochondrial activity, lipid metabolism, oxidative stress responses, and inflammatory pathways (Cao et al., 2016; Agbu and Carthew, 2021). Accordingly, they have been extensively implicated in metabolic diseases such as obesity and T2D (Agbu and Carthew, 2021). Noteworthy, the brain is unique among vertebrate tissues in expressing the largest number and greatest diversity of distinct miRNAs, where they play key roles in neural development, function, and plasticity (Cao et al., 2016; Motti et al., 2012). However, their specific contribution to metabolic regulation in neurological disorders has only recently gained attention as a distinct and highly relevant area of investigation. Importantly, dysregulated pattern of miRNAs signatures has been linked to metabolic disturbances in a broad range of brain disorders, from classic neurovegetative diseases such AD, or psychiatric disorders like SCZ, to brain cancer (Cao et al., 2016; Li et al., 2024; Peng and Croce, 2016).

## 2 MiR-7

**Figure 2:**
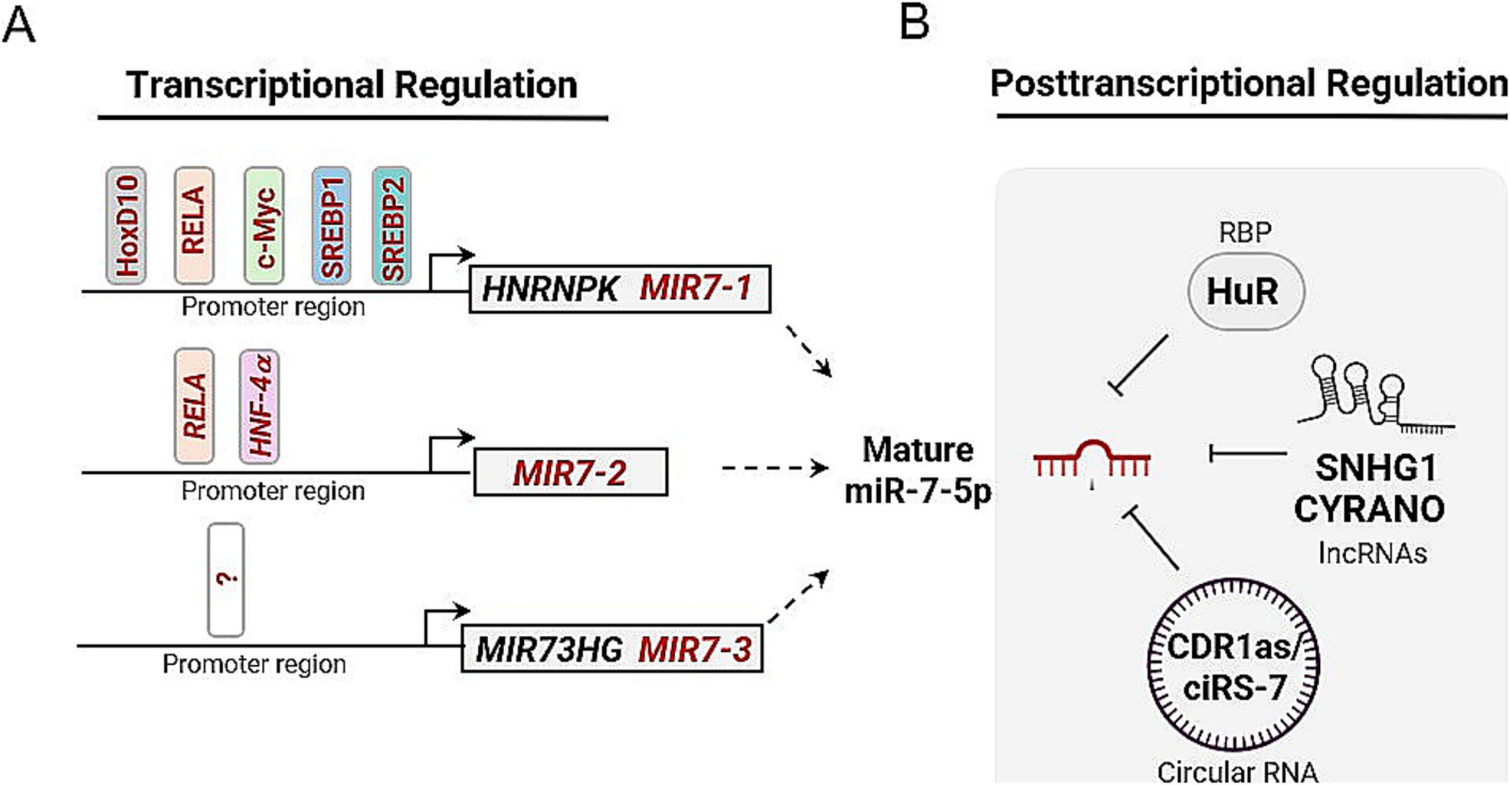
Transcriptional and posttranscriptional regulation of miR-7 expression. **(A)** Transcriptional regulation of the three human MIR7 loci. MIR7-1 is regulated by several transcription factors, including HOXD10, c-Myc, RELA, SREBP1, and SREBP2, whereas MIR7-2 is regulated by HNF4α and RELA. In addition, transcriptional activation of the HNRNPK host gene by SREBP1 and SREBP2 has been shown to coordinately induce the intronic MIR7-1 locus. The transcriptional regulation of MIR7-3 remains largely unexplored. **(B)** Post-transcriptional regulation of miR-7. MiR-7 biogenesis and activity are regulated by several modulators such as RNA-binding proteins (RBPs) and non-coding RNAs. The RBP HuR inhibits pri-miR-7 processing, whereas the circular RNA CDR1as/ciRS-7 and the lncRNA SNHG act as molecular sponges that sequester mature miR-7, thereby reducing its availability. In contrast, the lncRNA CYRANO promotes miR-7 degradation through a target-directed miRNA degradation (TDMD) mechanism, highlighting the complex multilayered regulation of miR-7 expression. Created with BioRender.com.

Among brain-enriched miRNAs, miR-7 has attracted particular attention because of its conserved expression pattern and its capacity to target multiple pathways relevant to neuronal metabolism, a broad regulatory capacity that links its dysregulation to neurodegenerative disorders such as PD and AD, as well as brain tumors like glioblastoma (GBM) (Fernández-de Frutos et al., 2019; Kushwaha et al., 2026; Torrecilla-Parra et al., 2025). MiR-7 is one of the most conserved miRNAs across species (Figure 1C). In humans there are three alleles (*MIR-7-1*, *MIR-7-2,* and *MIR-7-3*) within chromosomes 9, 15, and 19, respectively, that contribute to the same mature form of miR-7, similarly to mice (*miR-7a-1*, *miR-7a-2,* and *miR-7b*) (Figure 1B). Notably, *MIR-7-1* is strategically located in the last intron of the RNA binding protein heterogeneous nuclear ribonucleoprotein gene (*HNRNPK*) which facilitates the co-transcription of *MIR-7-1* with its host gene, suggesting potential coordinated actions (Fernández-de Frutos et al., 2019; Frutos et al., 2023; LaPierre et al., 2022). Interestingly, in humans, miR-7-3 is encoded within the long non-coding RNA (lncRNA) MIR7-3HG, which is highly expressed in the brain, although its functional roles are still not fully understood (Mao et al., 2023) (Figure 1B). In addition to this genomic organization, miR-7 expression is tightly regulated transcriptionally and posttranscriptionally at multiple stages of miRNA biogenesis (Frutos et al., 2023; Horsham et al., 2015; Chen et al., 2023). At the transcriptional level, the three MIR7 loci are regulated by multiple transcription factors in a locus- and context-dependent manner, including c-Myc, HOXD10, HNF4α and RELA (Reddy et al., 2008; Zhao X. D. et al., 2015; Ning et al., 2014). Furthermore, the intronic MIR7-1 locus may also be co-transcribed with its host gene *HNRNPK*, as demonstrated following SREBP1- and SREBP2-mediated activation of *HNRNPK* (Fernández-de Frutos et al., 2019; Frutos et al., 2023; LaPierre et al., 2022) (Figure 2A). At the posttranscriptional level, several layers of control have been described, including epigenetic processes such as DNA methylation, posttranscriptional regulation by RNA binding protein HuR (Srikantan et al., 2012) and by circular such as CDR1 (also known as ciRS-7), which act as biomolecular sponges for miR-7, sequestering it and modulating its activity (Chen et al., 2023; Hansen et al., 2013; Memczak et al., 2013). In addition, other lncRNAs such as SNHG (Cao et al., 2018) or CYRANO (Chen et al., 2023) have been shown to influence miR-7 expression (Figure 2B). Nevertheless, whether these miR-7 genes are differentially expressed in specific tissues, physiological or pathological conditions, and whether they contribute differently to the mature miR-7 pool, remains largely unknown.

Consistent with this complex regulation, miR-7 displays a characteristic tissue-specific expression pattern. Traditionally considered a neuroendocrine miRNA, miR-7 is highly abundant in several brain areas such as the cerebellum, cortex, substantia nigra, striatum (Junn et al., 2009), hippocampus and amygdala (Sanek and Young, 2012). However, its highest expression is found in the hypothalamus and the pituitary gland (LaPierre et al., 2022; Junn et al., 2009; Sanek and Young, 2012; Zacharjasz et al., 2024; Amar et al., 2012; Yuan et al., 2024; Herzer et al., 2012; Gao et al., 2019) which suggests a relevant role of miR-7 in the neurometabolic control of food intake and appetite (LaPierre et al., 2022; Sanek and Young, 2012; Zacharjasz et al., 2024; Amar et al., 2012; Yuan et al., 2024; Herzer et al., 2012), as well as other such as stress regulation and reward behavior (LaPierre et al., 2022; Sanek and Young, 2012), suggesting its potential role on other neuropsyquiatric disorders. Despite all this evidence, miR-7 functions have been traditionally explored on cancer, while brain metabolic functions and their impact on neurological disorders is a more recent area of investigation.

This review focuses on the current knowledge regarding the role of miR-7 in several neurological conditions, ranging from neurodegenerative diseases and neuropsychiatric disorders to brain tumors, and discusses the emerging evidence of its metabolic role in these pathologies. As summarized in Table 1, the strength of the available evidence varies considerably across these diseases, ranging from well-established molecular mechanisms to more preliminary or associative findings. Accordingly, the role of miR-7 should be interpreted in a disease- and context-dependent manner throughout this review.

**Table 1:** Summary of the current evidence supporting the role of miR-7 across neurological disorders.

Disease and level of evidence	Direction of miR-7 change	Validated targets	Metabolic/cellular processes affected	Expected therapeutic strategy	Model system used	Study
AD—well-established	↑	INSR, IGFR1, IRS2, AKT, IDE, LXR, ABCA1	Insulin signaling amyloidosis	Silencing miR-7 expression	Mouse neuroblastoma N2a cell line and BV-2 microglial cells 5x FAD and WT mice Postmortem human brains (area 9) controls and from patients with severe AD	Fernández-de Frutos et al. (2019)
	–	DHCR24, DHCR7, SC5D	Cholesterol homeostasis amyloidosis	Silencing miR-7 expression	N2a, SH-SY5Y, HEK293, Huh-7 and COS-7 cell lines 5x FAD, NPC1nmf164 and WT mice	Frutos et al. (2023)
PD—well-established	↓	α-syn	α-syn accumulation α-syn-mediated proteasome impairment oxidative stress	Using miR-7 as an α-syn inhibitor	MPTP-induced neurotoxin model of PD in HEK293, SH-SY5Y and NS20Y cells and in mice	Junn et al. (2009)
	–	α-syn	α-syn accumulation	Overexpression of miR-7 and miR-153	HEK293 cell line, primary cultures of rat or murine cortical neurons and astrocytes	Doxakis (2010)
	–	α-syn	α-syn accumulation	MiR-7 overexpression	AAV-miR-7 injected mice + inoculation with recombinant α-synuclein preformed fibrils	Zhang et al. (2021)
	↓	α-syn	AST reversed miR-7 inhibition and so affects α-syn accumulation and apoptosis	AST	SH-SY5Y cell line + MPP+ + AST	Shen et al. (2021)
	–	α-syn	α-syn accumulation m-TOR pathway Oxidative stress	Overexpression of miR-7 and miR-153	Murine cortical neurons + MPP+	Fragkouli and Doxakis (2014)
	–	NLRP3 α-syn	Inflammasome activation and downstream inflammatory response ↓ Microglial activation ↓ Neurodegeneration	Overexpression of miR-7	α-Syn-A53T over-expressed and Caspase-1 knockout mice microglial and neuronal primary cultures Murine BV-2 microglial cells	Zhou et al. (2016)
	-	NLRP3 α-syn	Inflammasome activation and downstream inflammatory response ↓ Microglial activation ↓ Neurodegeneration	Overexpression of miR-7	Adult neural stem cells derived from α-syn transgenic, caspase-1 knockout, double transgenic mice	Fan et al. (2016)
	–	VDAC1	Mitochondrial morphology and membrane potential maintenance Apoptotic cascade regulation	Overexpression of miR-7	SH-SY5Y neuroblastoma cells + MPP + VDAC1+/− mice + MPP+	Chaudhuri et al. (2016)
	–	TCF7L2	Dopaminergic neurons and oligodendroglial cell differentiation Wnt/β-catenin signaling	–	Human ESC line H9, and HEK 293 T	Adusumilli et al. (2020)
	↑	–	–	Biomarker	Serum samples from PD patients and healthy controls	Citterio et al. (2023)
	↑	–	–	Biomarker	CSF and plasma samples from PD, MSA and PSD patients, and healthy control	Starhof et al. (2019)
HD—emerging	↑	–	–	–	BACHD HD mouse model Primary neuronal culture	Olmo et al. (2021)
	↓	Tab2	Tab2-mediated TAK1–MKK4–JNK pro- apoptotic pathway	Restoring miR-7 expression	Human neuroblastoma SK-N-MC cells iPSC cultures Brain tissue samples from healthy controls and HD patients *Drosophila* lines HD model	Chen et al. (2025)
ALS—emerging	↑ in the beginning of the disease and then completely depleted	CCND1 proposed, not validated	Wnt/β-catenin pathway PI3K/AKT pathway	Using miR-7 as a target for future treatments	Blood samples from ALS patients, ALS-mimic, and controls	Gomes et al. (2023)
SMA—preliminary	↑ before the treatment ↓ after the treatment	–	–	Nusinersen	CSF samples from infants with SMA	D'Silva et al. (2023)
SCZ—emerging	↑	SHANK3	Synaptic plasticity	–	HEK293T and mouse hippocampal HT22 cell lines Plasma samples from SCZ patients and controls	Zhang et al. (2015)
	↑	–	–	Biomarker	Plasma and peripheral blood mononuclear cells of SCZ patients and controls	Sun et al. (2015a)
	↑	–	–	Biomarker	Plasma of SCZ patients and controls	Sun et al. (2015b)
	↑	–	–	Biomarker	Plasma of SCZ patients and controls	Song et al. (2014)
	–	–	Loop SNTG2-AS1/hsa-miR-7-5p/SLC7A5	–	*In silico* study of olphatory epithelium from SCZ patients	Sabaie et al. (2021)
	–	–	hsa-miR-7-3 hypomethylation	–	Postmortem brain tissue (BA9) from SCZ or BD patients	Zhao H. et al. (2015)
	–	SHANK3	SHANK3 interactome Cytoeskeletal and actin organization	–	Mice derived hippocampal neurons	Choi et al. (2015)
BD— Preliminary	↑	–	–	Biomarker	Plasma serum from BD-II patients	Lee et al. (2020)
	–	FARSB prposed, not validated	Indirect modulation of BDNF signaling	–	Blood samples from BD-II patients	Tsai et al. (2024)
GBM—Well-established	–	EGFR, upstream regulators of AKT pathway	EGRF inhibition AKT activation (glioma invasiveness)	Delivery of miR-7	Established, primary, and GSC lines	Kefas et al. (2008)
	–	RAF1	CircXPO1/miR-7-5p/RAF1 axis Tumor growth	Biomarker	Normal human astrocyte cell line (NHA) and human glioma cell lines (U87-MG, U251)	Wang et al. (2023)

↓	RAF1	Vascular endothelial cell proliferation inhibition	Using miR-7-5p as a guide for the antitumor angiogenesis drug development	Microvasculature from GBM or normal brain tissue derived from neurosurgeries	Liu et al. (2014)

↓	ENO2, STX17, SNAP29, BLOC1S4, SCARB2, AKT, mTOR	PI3K/AKT/mTORC1 signaling Glycolisis Oxidative stress Mitochondrial function Autophagy, and ECM remodeling	MiR-7 delivery	Mouse and human neuroblastoma cell lines (N2a and SH-SY5Y), monkey and human kidney cell lines (COS-7, HEK293), and the human glioblastoma U87-MG cell line GBM xenograft mouse model	Torrecilla-Parra et al. (2025)

–	FAK	MMP2/MMP-9 pathway ERK/AKT signaling GBM cell migration and invasion	MiR-7 as a potential therapeutic target for GBM intervention	Human glioma cell lines, U87, and U251 Human primary glioma samples	Wu et al. (2011)

–	TFF3	PI3K/AKT signaling GBM cell migration and invasion	Targeting this novel miR-7-5p/TFF3 axis may be a useful therapeutic strategy for GBMs	Human GBM cell lines U87MG, LN229, A172 and T98G (ATCC) Human glioma samples from patients	Shukla et al. (2018)

↓	STATB1	GBM cell migration and invasion	–	Human GBM cell lines U87 MG ATCC, U373 MG ATCC and NHAs Human glioma samples from patients	Yin et al. (2019)

↓	TBX2	Epithelial–mesenchymal transition and invasiveness	–	Human GBM cell lines, DBTRG-05MG (05 MG), G5T/ VGH (G5T), GBM8401 (8401), and GBM8901 (8901), and the SVG p12 astroglial cell line Tumor samples from patients	Pan et al. (2020)

↓	YY1	Cancer stem cell properties regulation	–	GBM tissue samples from patients TMZ-resistant cell line: GBM tumor cells LN229 Mouse xenograft model	Jia et al. (2019)

–	XIAP	Autophagy and intrinsic pathway of apoptosis regulation Migration and invasiveness	Luteolin + Silibinin + overexpression of miR-7-1-3p	Human glioblastoma U87MG (wild type p53) and T98G (mutant p53) cell lines Mouse xenograft model	Chakrabarti and Ray (2016)
BM—Emerging	↓	KLF4	MiR-7/KLF4 pathway Tumor adaptation within the neural microenvironment	–	Established breast cancer cell lines, metastatic variants, patient-derived breast cancer cell lines, primary tumor cells, CSCs, and non-tumor cell lines Mouse xenograft model	Okuda et al. (2013)
	↓	–	–	MiR-7-5p as a grading and prognostic biomarker	Tumor samples from patients with low grade glioma, high grade glioma, or BM from lung cancer	Nikolova et al. (2022)

The table summarizes the direction of its dysregulation, validated direct targets, affected pathways, proposed therapeutic strategies, experimental models, and current level of evidence. Evidence was classified as *well-established* (several independent studies with validated molecular mechanisms), *emerging* (limited or incomplete mechanistic evidence), or *preliminary/associative* (predominantly associative observations or few mechanistic studies requiring further validation).

## 3 Neurodegenerative diseases

### 3.1 Alzheimer’s disease

Neurodegeneration is defined as the biological process characterized by the progressive loss of neuronal structure and function, ultimately leading to neuronal death (Bushati and Cohen, 2008). Among neurodegenerative diseases, AD is the most common. To date, it is estimated that around 32 million people have AD dementia, while nearly 400 million may be in a preclinical stage of the disease (Gustavsson et al., 2023). It is clinically characterized by progressive memory impairment, which is linked to the presence of two main pathological hallmarks: extracellular amyloid-β (Aβ) plaques and intracellular neurofibrillary tangles (NFTs) (Soria Lopez et al., 2019). Both structures precede the neuronal death and brain degeneration observed at later stages of the disease. The pattern of Aβ plaque deposition starts to accumulate in the cortex, which potentially affects consciousness, and later spreads to other cortical regions, the hippocampus, basal ganglia, and midbrain. On the other hand, NFTs tend to accumulate initially in the locus coeruleus, a brainstem region involved in arousal and attention, before spreading through the hippocampus and cortex, and reaching cortical areas responsible for higher cognitive and executive functions (Ávila-Villanueva et al., 2022; Braak and Del Tredici, 2011). Collectively, these alterations could potentially justify other symptoms such as cognitive disturbance, disruptive episodic memory, disorientation, delusions, language decline, and other behavioral alterations (Soria Lopez et al., 2019). While early-onset AD is caused by mutations in the *APP* and *PSEN* genes, which promote Aβ accumulation (Dai et al., 2017), the mechanisms underlying elevated brain Aβ levels in most sporadic late-onset AD cases remain unclear (Sarma and Chatterjee, 2024). Particularly noteworthy is the growing body of experimental and epidemiological evidence linking diabetes to the development of AD, to the extent that AD associated with brain insulin resistance (IR) has been proposed as “Type 3 Diabetes” (Michailidis et al., 2022). In this line, IR and alterations in cholesterol metabolism, mitochondrial dysfunction, and oxidative stress represent key pathophysiological features shared by AD and diabetes (Christodoulou et al., 2026; Rudajev and Novotny, 2022; Zhou Q. et al., 2023; Kolb et al., 2023). These disorders exhibit overlapping pathogenic mechanisms that impair cognitive function, largely driven by these processes, and closely linked to Aβ-associated pathology in AD (Kellar and Craft, 2020). In the CNS insulin, insulin-like growth factor (IGFs) and their receptors (INSR, IGFRs) play a crucial role in learning and memory, regulating processes such as neuronal stem cell activation, cell growth, synaptic maintenance as well as Aβ degradation (Martín-Martín et al., 2022). One of the key metabolic pathways involved in these processes is the PI3K–AKT–mTOR signaling pathway, which is disrupted in AD and contributes to an imbalance between autophagy and apoptosis, thereby promoting neurotoxicity (Kumari et al., 2023; Pan et al., 2024). Upon binding of insulin or IGFs to their receptors, adaptor proteins such as IRS2 become phosphorylated and activate PI3K, which subsequently leads to phosphorylation and activation of AKT. AKT promotes the activation of mTOR signaling and phosphorylates downstream targets such as GSK-3β, thereby inhibiting its activity. As GSK-3β promotes tau hyperphosphorylation and its aggregation into NFTs (Pan et al., 2024), AKT activation and the consequent inhibition of GSK-3β may reduce cognitive impairment and neuronal dysfunction by limiting apoptosis (Kumari et al., 2023; Pan et al., 2024; Emamian, 2012). However, during AD progression, Aβ oligomers can activate GSK-3β, which in turn suppresses PI3K/AKT/mTOR signaling and promotes tau hyperphosphorylation, enhancing amyloid plaque formation, apoptosis, and neuroinflammation, ultimately contributing to the neurodegenerative phenotype (Kumari et al., 2023; Pan et al., 2024). Importantly, Aβ has been shown to bind to INSR, competing with insulin itself. This reduces receptor activation and downstream signaling through pathways such as PI3K/AKT, which creates a positive feedback loop where more Aβ leads to less insulin signaling, and consequently promotes even more Aβ accumulation and amplifies tau pathology (Zhao et al., 2008). Additionally, one of the downstream targets of mTORC1 is SREBP2, a transcription factor that regulates genes involved in cholesterol biosynthesis. Among these enzymes, cholesterol 24-dehydrocholesterol reductase (*DHCR24*), also known as SELADIN (Selective Alzheimer Disease Indicator) and is found to be progressively downregulated during aging and the development of AD (Bai et al., 2022). In this metabolic context, miR-7 has been shown to act as a key regulator of the crosstalk between neurodegeneration and metabolic decline. Elevated levels of miR-7 in AD (Fernández-de Frutos et al., 2019) may contribute to reduced activation of the PI3K/AKT/mTOR pathway, thereby influencing the intricate metabolic interplay underlying diabetes and AD pathophysiology. In line with this, Fernández-de Frutos et al. (2019) have shown that miR-7 targets essential regulators of insulin signaling pathway and homeostasis, such as *INSR*, *IGFR1*, *IRS2*, and *AKT*. Additionally, this study showed that miR-7 also suppresses the expression of the insulin-degrading enzyme (*IDE*), an important target in AD that degrades both insulin and Aβ *in vitro* and *in vivo*. Consistent with these effects, the researchers demonstrated that miR-7 overexpression increases Aβ levels and correlates with its significant upregulation in the brain of obese mice as well as in the brains of human AD patients (Fernández-de Frutos et al., 2019). Besides the insulin signaling pathway, they probed miR-7 blocks other neuroprotective pathways by inhibiting the expression of liver X receptor (LXR) and its transcriptional target ATP-binding cassette transporter (*ABCA1*), which promotes ApoE lipidation and reduced Aβ accumulation (Fernández-de Frutos et al., 2019). In line with these observations, another study demonstrated that miR-7 inhibits cholesterol synthesis through direct posttranscriptional regulation of *DHCR24* (Frutos et al., 2023). Notably, miR-7 expression gradually increases in the cortex of 5xFAD AD mouse model during the progression of neurodegeneration and Aβ accumulation and is inversely correlated with the levels of DHCR24 and IDE mRNAs, consistent with its role in impairing both cholesterol homeostasis and insulin/Aβ metabolism. Collectively, these studies support a central role for miR-7 in coupling metabolic regulation with neurodegenerative pathology, through coordinated effects on lipid homeostasis and insulin signaling, thereby integrating metabolic control and neuroprotective mechanisms (Frutos et al., 2023). Thus, reducing miR-7 expression during AD may offer a promising therapeutic strategy to mitigate metabolic dysfunction and cognitive decline.

### 3.2 Parkinson’s disease

PD is the second most common neurodegenerative disease after AD, with a prevalence of approximately 8.5 million people worldwide (Li et al., 2025). PD often presents initially with non-motor symptoms, such as sleep disturbances, gastrointestinal dysmotility, anxiety, depression, dementia, and cognitive impairment. As the disease progresses, the classical triad of motor symptoms, tremor, rigidity, and bradykinesia gradually emerges. This clinical picture reflects the progressive loss of dopaminergic neurons in the substantia nigra pars compacta (SNpc), whose projections to the striatum are essential for motor control. This neurodegenerative process is accompanied by intracellular *α*-synuclein (α-syn) aggregation leading to the formation of Lewy bodies (LB) (Kouli et al., 2018).

Although PD is primarily multifactorial, monogenic familial forms have been described, including autosomal dominant cases linked to mutations in *α*-syn gene (*SNCA*) and autosomal recessive forms associated with mutations in genes coding Parkin RBR E3 ubiquitin protein ligase (*PRKN*), PTEN-induced putative kinase 1 *(PINK1)* or Parkinsonism Associated Deglycase (*DJ-1/PARK7*), among others. These genes play critical roles in mitochondrial quality control, oxidative stress response, and protein homeostasis, highlighting the role of metabolic processes in the neurodegenerative mechanisms underlying PD (Kouli et al., 2018; Su et al., 2026). *α*-Syn is present in the cytosol, mitochondria, and nucleus, where it serves as a chaperone influencing synaptic vesicle turnover, intracellular trafficking, and mitochondrial homeostasis (Su et al., 2026). In PD, genetic factors, ageing, and environmental influences promote *α*-syn aggregation into insoluble fibrils, leading to mitochondrial impairment in vulnerable dopaminergic neurons (Wang et al., 2019). These aggregates interact with mitochondria, impairing oxidative phosphorylation (Dong-Chen et al., 2023). Thus, there is a reduction in ATP production and fragmentation of mitochondria, which generate energy deficits and oxidative stress that further accelerate *α*-syn fibrillization (Dong-Chen et al., 2023; Moon and Paek, 2015; Sian-Hulsmann et al., 2024). In addition to these effects, mitochondrial dysfunction can also impair protein degradation systems, including the ubiquitin–proteasome system and autophagy–lysosomal pathway, ultimately contributing to dopaminergic neuron loss (Moon and Paek, 2015).

In line with mitochondrial alterations, α-syn accumulation in dopaminergic neurons disrupts dopamine storage, promoting neurotoxicity. Dopamine can undergo auto-oxidation, leading to the formation of reactive oxygen species (ROS) and highly reactive dopamine-quinones (DAQs) (Zhou Z. D. et al., 2023). These neurons also exhibit increased autophagic stress, partly due to impaired trafficking and defective lysosomal clearance. This leads to the accumulation of damaged cellular components, which are commonly found within LBs (Rademacher and Nakamura, 2024).

Collectively, these alterations disrupt brain energy homeostasis in PD, particularly in highly connected, synapse-rich regions that support higher-order functions. Importantly, *α*-syn contributes to severe brain hypometabolism, reflected by reduced glucose utilization, likely arising from its combined effects on mitochondrial dysfunction, synaptic impairment, and neuronal loss, and is associated with cognitive decline (Westphal and Chandra, 2013; Firbank et al., 2017; Devrome et al., 2019). Furthermore, *α*-syn is a lipid-binding protein that associates with cellular and subcellular membranes, including lipid rafts, and interacts dynamically with membrane lipids (Fortin et al., 2004; Emanuele et al., 2016). Its deficiency or mutation can compromise membrane integrity, and disrupt intracellular signaling, further exacerbating metabolic dysfunction (Sian-Hulsmann et al., 2024; Westphal and Chandra, 2013; Maltseva et al., 2024). As proposed for AD, miR-7 emerges as a key neurometabolic regulator at the interface between *α*-syn aggregation and metabolic dysfunction in PD. In fact, it has been demonstrated that miR-7 directly targets α-syn mRNA resulting in significant decreases in both transcript and protein levels *in vitro* and *in vivo*, rising clues on the potential neuroprotective role of miR-7 on PD (Junn et al., 2009; Doxakis, 2010; Zhang et al., 2021; Shen et al., 2021). In this context, downregulation of α-syn may protect neurons from the harmful effects of its accumulation, such as oxidative stress (Junn et al., 2009; Zhang et al., 2021). Fragkouli and Doxakis (2014) reported that miR-7 overexpression protects against neurotoxicity and oxidative stress in an *in vitro* cortical model of PD. Specifically, the authors propose that miR-7 restores neuronal viability by activating mTORC1 signaling, increasing the expression of the anti-apoptotic protein Bcl-2, and consequently inhibiting caspase-3 activation. However, this interpretation contradicts the expected effect, as mTOR is a direct target of miR-7 and would therefore be predicted to be downregulated (Xu et al., 2017; Wang et al., 2017). One possible explanation is that miR-7 with other factors indirectly modulate mTORC1 signaling in this disease context as an adaptive mechanism, for example by downregulating upstream inhibitors, resulting in a net activation of pro-survival pathway. Consistent with this possibility, it has been shown that during hypoxia miR-7 directly represses the stress-responsive protein REDD1, a well-established negative regulator of mTORC1, thereby relieving REDD1-mediated inhibition and promoting mTOR signaling (Seong et al., 2019). These observations suggest that the biological outcome of miR-7 on mTOR activity may depend on the cellular context and the balance between its direct and indirect molecular targets. Such indirect regulatory effects are common in miRNA-mediated networks (Kar et al., 2021), although further studies are needed to decipher if similar mechanism operates in the PD model described (Fragkouli and Doxakis, 2014).

Growing interest is focused on identifying miR-7 modulators as potential therapeutic strategies for PD. In this context, Shen et al. (2020) published that the neuroprotective effects of the antioxidant astaxanthin (AST) are linked to the miR-7/SNCA axis in both *in vivo* and *in vitro* models. MiR-7 directly targets *α*-syn mRNA, limiting the accumulation of misfolded proteins and promoting neuronal survival. These authors suggest that AST treatment reversed the effects of miR-7 knockdown, increasing Bcl-2 levels while reducing Bax and cleaved caspase-3 levels in the presence of a neurotoxin that models PD, further supporting its anti-apoptotic and neuroprotective effects (Shen et al., 2021). In addition to these effects, miR-7 also seems to exert neuroprotective actions by attenuating neuroinflammation. Notably, the microglial nod-like receptor protein-3 (NLRP3) is a direct target of miR-7, and its repression limits inflammasome activation and downstream inflammatory responses (Zhou et al., 2016; Fan et al., 2016). Neuroinflammation in PD is partly driven by *α*-syn, which promotes microglial NLRP3 inflammasome activation following its internalization, lysosomal stress, and ROS production (Codolo et al., 2013; Gordon et al., 2018). Accordingly, miR-7 may reduce neuroinflammation both directly, through NLRP3 repression, and indirectly, by limiting α-syn accumulation. Consistent with this, in PD mouse models, stereotaxic administration of miR-7 mimics attenuates dopaminergic neurodegeneration and reduces microglial activation (Zhou et al., 2016; Fan et al., 2016). MiR-7 may also protect neurons from neurotoxicity and neuroinflammation by preserving mitochondrial integrity. Chaudhuri et al. (2016) reported that miR-7 maintains mitochondrial morphology and membrane potential, and regulates the expression of mitochondrial proteins, including voltage-dependent anion channel 1 (VDAC1), a key component of the mitochondrial permeability transition pore (mPTP). These authors showed that by downregulating VDAC1, miR-7 reduces mPTP opening, preserves mitochondrial membrane potential, and prevents activation of the apoptotic cascade (Chaudhuri et al., 2016). This beneficial effect may be explained by the proposed role of VDAC as a pathway for *α*-syn entry into mitochondria (Rostovtseva et al., 2015). Consistently, reduced VDAC1 levels have been associated with decreased α-syn localization at the inner mitochondrial membrane and, consequently, with reduced mitochondrial damage in PD (Rovini et al., 2020). However, these observations differ from that described in other neurodegenerative context such as AD, where both miR-7 and VDAC1 have been reported to be increased, suggesting that the expected inverse miR-7-VDAC1 regulatory relationship is not maintained in this pathological context, These findings further support the context-dependent role of miR-7 in mitochondrial regulation across neurodegenerative diseases, although the molecular basis underlying these disease-specific differences remains to be elucidated.

Beyond its metabolic and protective roles, miR-7 also contributes to the differentiation of dopaminergic neurons and oligodendroglial cell fates. A study in zebrafish and human cell cultures demonstrated that miR-7 acts as a negative regulator of Wnt/ *β*-catenin signaling by targeting the Wnt transducer TCF7L2 and potentially through indirect modulation of the key neural fate regulator Sonic hedgehog (Shh). This regulatory interplay influences the balance between dopaminergic neurons and glial cells during neural progenitor differentiation (Adusumilli et al., 2020).

MiR-7 is also involved in broader metabolic regulation, including glucose and lipid homeostasis. Evidence suggests that downregulation of the PI3K/AKT/mTOR pathway in PD could be protective by promoting autophagy and reducing neuroinflammation (Wang et al., 2024; Pan et al., 2009). Although no study has directly investigated the relationship between miR-7 and this pathway in PD, it is known that AKT and other proteins activating this signaling cascade are targets of miR-7 (Fernández-de Frutos et al., 2019).

Besides its functional effects in PD, several studies support a role for miR-7 as a biomarker. For instance, circulating miR-7-1-5p levels in the serum of PD patients have been found to be increased compared to healthy controls and to correlate with *α*-syn levels, suggesting it could help identify PD patients and reflect disease changes. Although miR-7 directly targets α-syn, this positive association may reflect a compensatory response, in which miR-7 is upregulated in the blood in an attempt to counteract increased α-syn expression (Citterio et al., 2023). In the cerebrospinal fluid, miR-7-1-5p has also been detected as a biomarker of parkinsonian syndromes; however, no association was observed with α-syn levels (Starhof et al., 2019). Notably, other parkinsonian syndromes such as Multiple System Atrophy (MSA), a neurodegenerative synucleinopathy, and Progressive Supranuclear Palsy (PSP), a tautopathy also exhibit elevated miR-7 levels. In particular, miR-7-5p was significantly upregulated in the cerebrospinal fluid of patients with PD, MSA, and PSP compared with controls and emerged as one of the most discriminative miRNAs for distinguishing synucleinopathies from healthy individuals (Starhof et al., 2019). This increase in miR-7 levels may represent a temporally restricted protective response to neurodegeneration, although it may become insufficient as the disease progresses.

Overall, literature describes miR-7 as a neuroprotective factor in the context of PD, through protecting neurons from neurotoxicity and neuroinflammation, while preserving mitochondrial integrity by interfering with *α*-syn deposits. Therefore, in PD, enhancing miR-7 expression could offer a promising therapeutic strategy by mitigating α-syn accumulation and the consequent pathogenic processes.

### 3.3 Huntington’s disease

HD is an autosomal dominant neurodegenerative disorder with a global prevalence of approximately 4.88 cases per 100,000 individuals (Medina et al., 2022). It is caused by an expansion of CAG trinucleotide repeats in the huntingtin (*HTT*) gene, which is essential for normal embryonic development, neuronal survival, and intracellular trafficking (Saudou and Humbert, 2016; Schultz et al., 2023). This mutation results in an expanded polyglutamine tract in the huntingtin protein, promoting protein misfolding, aggregation, and neuronal toxicity (Schultz et al., 2023; Ratovitski et al., 2012; Walker, 2007; Olmedo-Saura et al., 2025). Disease risk and earlier onset correlate with increasing CAG repeat length (Schultz et al., 2023; Walker, 2007; Olmedo-Saura et al., 2025). The primary neuropathological feature of HD is the selective degeneration of the striatum (Schultz et al., 2023; Walker, 2007), particularly the caudate and putamen (Walker, 2007). Medium spiny neurons are predominantly affected, leading to disruption of basal ganglia circuitry (Walker, 2007). Clinically, HD is characterized by a triad of motor disturbances, including chorea and bradykinesia, cognitive decline affecting language, executive function, memory and attention, as well as behavioral or psychiatric symptoms such as apathy, anxiety, depression, and psychosis, with progressive worsening over time (Singh and Agrawal, 2022; Schultz et al., 2023; Walker, 2007; Olmedo-Saura et al., 2025). Beyond neurological symptoms, metabolic impairments are evident in multiple organs and tissues, supporting the view that this is a multisystem disorder with a strong metabolic basis. For instance, weight loss independent of caloric intake, and consequently cachexia is a recognized feature of HD. Additional systemic effects of HD both pre-symptomatic and symptomatic patients include skeletal muscle atrophy, cardiac dysfunction, and disrupted glucose regulation (Singh and Agrawal, 2022). These changes are accompanied by disturbances in key metabolic pathways, affecting glucose and insulin signaling, lipid metabolism, and circulating factors such as leptin, ghrelin and branched-chain amino acids, as well as intermediates of the tricarboxylic acid (TCA) cycle, electron transport chain, and glycolysis (Singh and Agrawal, 2022). At the cellular level, different mechanisms have been proposed to underlie these metabolic alterations, including reduced glycolytic activity, impaired Krebs cycle function, defective oxidative phosphorylation, altered mitochondrial calcium handling, or oxidative stress (Singh and Agrawal, 2022). Several studies have shown that miRNA expression profiles are altered in HD (Olmo et al., 2021; Dong and Cong, 2021). In fact, the overexpression pattern of specific miRNAs with therapeutic potential has been found (Dong and Cong, 2021). With regard of miR-7 in HD, research is still at early stages. Olmo et al. (2021) identified miR-7-a-5p and miR-7-a-2-3p upregulation in the striatum of a mouse model that represents the presymptomatic and symptomatic phases of HD, respectively. However, the inhibition of miR-7 *in vitro* did not produce a significant effect on neuronal cell death compared to basal levels (Olmo et al., 2021), indicating that the results remain inconsistent. A recent study reported a significant reduction in miR-7-5p levels in postmortem striatal tissue from HD patients. In this context, the transcription factor Yin Yang 1 (YY1) is sequestered by mutant CAG RNA foci and polyglutamine protein aggregates, preventing it from repressing its target gene, poly(A) RNA polymerase D5 (PAPD5). As a consequence, PAPD5 is upregulated and promotes the adenylation and degradation of miR-7-5p. Under normal conditions, miR-7-5p targets TAK1 binding protein (Tab2), a key regulator of pro-apoptotic signaling that activates the TAK1 cascade, that ultimately leading to caspase-3 activation and neuronal cell death. Therefore, reduced miR-7-5p levels may contribute to neurodegeneration in HD (Chen et al., 2025).

Furthermore, several reports have shown that AKT signaling is altered in HD brain patients, especially in the striatum (Humbert et al., 2002; Colin et al., 2005). As a substrate of AKT, huntingtin requires phosphorylation to mediate the neuroprotective effects of IGF-1 and reduce the formation of intranuclear inclusions of polyglutamine-expanded huntingtin (Humbert et al., 2002). Given that AKT is also a target of miR-7 (Fernández-de Frutos et al., 2019), its dysregulation in HD may influence miR-7-dependent pathways, potentially affecting cellular metabolism and survival. However, to date, no studies have directly explored this relationship, and the role of AKT in HD remains controversial.

Overall, the available evidence regarding miR-7 expression in HD remains inconsistent. The apparently contradictory findings are likely attributable to differences between experimental mouse models and postmortem human brain samples, reflecting species-specific regulation of miR-7 together with differences in disease stage, as animal models usually recapitulate early or preclinical phases of disease, whereas human postmortem tissues predominantly represent advanced pathological stages. Based on the currently available human data, restoration of physiological miR-7 levels could potentially attenuate neuronal cell death by repressing the pro-apoptotic Tab2–TAK1–MKK4–JNK signaling pathway. However, given that this conclusion is supported by a single study, further validation is required before therapeutic implications can be drawn.

### 3.4 Motor neuron diseases

Motor neuron diseases (MNDs) are a heterogeneous group of progressive neurodegenerative disorders characterized by the degeneration of upper and/or lower motor neurons (MNs) leading to muscle weakness, paralysis, and respiratory failure. This category includes conditions such as amyotrophic lateral sclerosis (ALS), spinal muscular atrophy (SMA), and Spinal and Bulbar Muscular Atrophy (SBMA) (Barone and Qi, 2023). In motor neurons, miRNAs are crucial for development, function, plasticity, and maintenance (Hawley et al., 2017).

*ALS* is the most common MND in adults (Gwathmey et al., 2023) with an estimated worldwide prevalence ranging from 4.1 to 8.4 per 100,000 people (Longinetti and Fang, 2019). Beyond MN degeneration, ALS is increasingly recognized as a network disorder. Structural connectivity is consistently reduced, particularly in corticospinal and extra-motor tracts. Functional connectivity changes are more heterogeneous, with evidence of early hyperexcitability and network reorganization, followed by progressive disruption as the disease advances (Fortanier et al., 2019). The degeneration of MNs in the motor cortex, brainstem nuclei, and anterior horn of the spinal cord leads to progressive muscle detriment. It often begins in a focal region but gradually spreads to other areas, including the respiratory muscles, and is frequently accompanied by non-motor symptoms such as behavioral changes, executive dysfunction, and language impairments (Masrori and Van Damme, 2020). Similarly to other neurodegenerative diseases, ALS is thought to result from a combination of genetic factors, environmental influences, and age-related dysfunction. To date, more than 40 genes have been associated with the disease, many of which are linked to protein aggregation, RNA metabolism, oxidative stress, cytoskeletal dynamics, axonal transport, and neuroinflammation (Feldman et al., 2022). MN degeneration is typically accompanied by astrogliosis and microgliosis, as well as TAR DNA-binding protein 43 (TDP-43) inclusions in surviving neurons. TDP-43 is an RNA- and DNA-binding protein primarily located in the nucleus, while its nuclear depletion and cytoplasmic aggregation constitute a hallmark of ALS (Masrori and Van Damme, 2020). Pathological TDP-43 disrupts proteostasis and autophagy, processes further compromised by mutations in autophagy-related genes such as sequestosome 1 (*SQSTM1*, which encodes the protein p62). These alterations lead to the accumulation of toxic protein aggregates and dysfunctional mitochondria, promoting cellular stress and neurodegeneration. In addition, mutant p62 can accumulate and dysregulate signaling pathways, including inflammation, thereby exacerbating disease progression (Masrori and Van Damme, 2020; Feldman et al., 2022; Evans and Holzbaur, 2019). Mutations in *SOD1* represent another well-established genetic cause of ALS and are associated with protein aggregation, mitochondrial dysfunction, and increased oxidative stress (Masrori and Van Damme, 2020; Feldman et al., 2022). Consistent with these molecular alterations, ALS is also characterized by systemic metabolic disturbances, including hypermetabolism and increased cellular turnover in response to neurodegeneration (Nelson and Trotti, 2022; Dupuis et al., 2011). Most patients exhibit a hypermetabolic state, which contributes to progressive nutritional decline and is associated with poor prognosis (Dupuis et al., 2011; Fayemendy et al., 2021). Evidence from animal models expressing mutant TDP-43 or SOD1 indicates that impaired muscle energy homeostasis is an intrinsic feature of the disease (Dupuis et al., 2011). Although the underlying mechanisms remain unclear, they likely involve increased glucose and lipid utilization, mitochondrial dysfunction, and altered hypothalamic regulation (Nelson and Trotti, 2022).

In an effort to identify reliable plasma biomarkers, Gomes et al. (2023) profiled several miRNAs and found that miR-7-2-3p was significantly overexpressed in ALS patients. Notably, it was the only miRNA that showed significant differences compared to ALS-mimic disorders, in which it was undetectable. Consistent with a potential role in ALS pathophysiology, pathway enrichment analysis suggested that miR-7-2-3p may downregulate *CCND1* (Gomes et al., 2023), which encodes Cyclin D1, a key regulator of the cell cycle and a downstream target of the Wnt/β-catenin pathway (Tetsu and McCormick, 1999; Chen et al., 2012), thereby promoting glial proliferation in the spinal cord (Chen et al., 2012; Adusumilli et al., 2020). Given that glial proliferation is a hallmark of ALS pathology (Feldman et al., 2022; Chen et al., 2012), this early upregulation may reflect a protective response to neurodegeneration, with levels declining as the disease progresses (Gomes et al., 2023). However, as patients were not necessarily recruited at early stages, variability in disease progression may influence these observations. In addition, the PI3K/AKT pathway is downregulated in ALS patients and in SOD1 mutant mouse models, leading to impaired immune cell proliferation in response to cytokine stimulation (Gomes et al., 2023). Although not directly linked in ALS, this pathway is a known target of miR-7 in other contexts (Fernández-de Frutos et al., 2019; Torrecilla-Parra et al., 2025; Mao et al., 2023), suggesting that miR-7 may also contribute to its modulation in ALS.

*SMA* is an autosomal recessive disease that affects 1 in every 10.000 neonates, which usually have mutations on the survival motor neuron (*SMN*) gene. SMN protein is ubiquitously expressed and participates in several essential cellular processes. However, spinal MNs are particularly vulnerable to decreased SMN levels, and the mechanisms behind this selective susceptibility remain largely unknown (Edens et al., 2015). Clinically, it often affects proximal leg muscles, while the upper extremities are largely spared (Barone and Qi, 2023; Russman, 2007). An approved treatment is the antisense oligonucleotide Nusinersen, which is administered intrathecally and modulates the splicing of the *SMN2* gene, promoting the prevention of neuronal loss (Claborn et al., 2019). Since the molecular mechanisms underlying the therapeutic response to this drug are not fully understood, a study was conducted to evaluate the miRNA profile in children with SMA treated with Nusinersen. The results identified several differentially expressed miRNAs, among which miR-7-5p stood out, showing significantly lower levels compared to before the start of treatment (D'Silva et al., 2023). Nonetheless, the neuroprotective effect of miR-7 may emerge over a longer period of treatment, aligning with functional recovery (D'Silva et al., 2023).

*SBMA*, also known as Kennedy’s disease, is an X-linked recessive disease that affects 1–2 individuals per 100,000 globally (Wilton-Clark et al., 2023). It is caused by a CAG trinucleotide repeat in exon 1 of the androgen receptor (*AR*). The molecular mechanisms of SBMA are not yet fully understood. However, current evidence suggests that protein misfolding, aggregation, impaired proteostasis, mitochondrial dysfunction, and altered autophagy are important pathogenic features (Katsuno et al., 2012). In SBMA, transcription factor E (TFEB) activity and its downstream target genes (e.g., *LC3*, *Vps11*, *Vps18*, and *Lamp1*) are upregulated in skeletal muscle in both mouse models and in patient tissue (Rodríguez-Muela, 2020; Rusmini et al., 2015), although contrasting findings have been found in iPSC-derived MNs from SBMA patients, as TFEB overexpression restored the autophagic flux defect (Cortes et al., 2014), indicating that its role in SBMA remains unclear and may vary depending on the tissue and cell type (Rodríguez-Muela, 2020). Dysregulated or differentially expressed miRNAs have been identified in SBMA, and miRNA-based therapeutic approaches, including AAV-mediated delivery such as miR-196a, have shown disease-modifying effects in mouse models by targeting AR mRNA and ameliorating disease phenotypes (Miyazaki et al., 2012). The absence of studies on miR-7 in MNs from SBMA patients prevents firm conclusions about its role in the disease, and its lack of evaluation in patient tissues or plasma also precludes any assessment of its biomarker potential. However, miR-7 may still participate in disease-related pathways, influencing TFEB levels and autophagic activity, as suggested by its ability to influence these pathways and upregulate TFEB in other cell types such as GBM (Torrecilla-Parra et al., 2025). Similarly, this could be extended to other MNDs, where metabolic regulation, autophagy, and mitochondrial quality control are commonly impaired (Rodríguez-Muela, 2020; Rusmini et al., 2015).

## 4 Neuropsychiatric disorders

### 4.1 Schizophrenia

Psychiatric disorders are multifactorial conditions arising from the interaction between genetic predisposition and environmental vulnerability (Fernández-Castillo and Martín-García, 2022). Among these, SCZ affects approximately 23 million people worldwide and, despite not being the most prevalent mental disorder, accounts for nearly 50% of psychiatric hospitalizations (World Health Organization, 2025). Clinically, SCZ is characterized by psychotic symptoms, including delusions and hallucinations, disorganized behavior, negative symptoms such as emotional blunting and social withdrawal, motor disturbances, and cognitive impairment (World Health Organization, 2025; Shen et al., 2023; Owen et al., 2016). At the neurobiological level, structural brain alterations appear to precede clinical onset, affecting both gray and white matter and progressively worsening over time. As the disorder advances, these changes extend to regions involved in cognition, emotion, and sensory processing, and are accompanied by disrupted functional connectivity and reduced white matter integrity, suggesting impaired communication between brain areas (Shen et al., 2023; Owen et al., 2016). Although SCZ is highly heritable, its genetic architecture is complex and polygenic, with most variants conferring modest risk (Owen et al., 2016). Notably, genes related to extracellular matrix (ECM) organization and cell adhesion, including collagen-related genes, have been implicated, pointing to alterations in brain structural integrity (Owen et al., 2016; Unzueta-Larrinaga et al., 2026; Su et al., 2017). In parallel, several risk genes converge on synaptic function, particularly glutamatergic signaling. For instance, *GRIN2A*, encoding a subunit of the NMDA receptor, and *SHANK3*, a key scaffolding protein at the synapse, have been associated with synaptic dysfunction and dendritic spine abnormalities (Owen et al., 2016; Huang et al., 2023). These findings support the glutamatergic hypothesis as a central framework for SCZ (Hu et al., 2015). However, additional models have been proposed. The dopaminergic hypothesis implicates dysregulated dopamine signaling (Brisch et al., 2014), while more recent evidence points to metabolic alterations involving disrupted astrocyte–neuron coupling. Impairment of the lactate shuttle may lead to neuronal energy deficits, ultimately affecting synaptic activity and glutamatergic neurotransmission (Roosterman and Cottrell, 2021).

Growing evidence points to epigenetic dysregulation, particularly involving miRNAs, as a contributing mechanism in SCZ (Li et al., 2024; Smigielski et al., 2020). Within this context, miR-7 has been repeatedly found upregulated in blood and brain samples of SCZ patients (Smigielski et al., 2020; Zhang et al., 2015; Sun et al., 2015a; Sun et al., 2015b; Song et al., 2014; Beveridge et al., 2010; Liu X. et al., 2024; Kim et al., 2010; Ghafouri-Fard et al., 2021), as well as in the olfactory epithelium (Sabaie et al., 2021). This increase has been linked to hypomethylation of hsa-miR-7-3, suggesting an epigenetic component to its dysregulation (Zhao H. et al., 2015). Together, these observations support miR-7 as a potential biomarker in SCZ. At the functional level, miR-7 may contribute to disease-related mechanisms through the regulation of synaptic genes. In particular, it downregulates *SHANK3* (Zhang et al., 2015; Choi et al., 2015), altering its expression and affecting dendritic spine structure, and may also impact actin-associated pathways interacting with SHANK3 (Choi et al., 2015). Besides, miR-7 may also modulate key signaling pathways implicated in SCZ such as the Akt family (Akt1–3), which is involved in synaptic signaling and neuronal survival, with Akt1 showing the strongest genetic association with SCZ (Emamian, 2012). Among SCZ-related signaling pathways involving Akt1 (Emamian, 2012; Mizuki et al., 2021), the Akt–GSK3 and Wnt/β-catenin pathways have been most strongly associated with miR-7 regulation in other contexts (Torrecilla-Parra et al., 2025; Su et al., 2022; Pei et al., 2022; Liu C. et al., 2024). Collectively, miR-7 is consistently upregulated in SCZ and may contribute to disease pathophysiology by modulating synaptic and signaling pathways, supporting its potential as both a biomarker and regulatory factor, although its precise role remains to be established.

### 4.2 Bipolar disorder

Bipolar disorder (BD) is a chronic psychiatric condition characterized by recurrent episodes of mania or hypomania, depression, and mixed states that significantly impair daily functioning. It is classified into BD-I, defined by at least one manic episode, and BD-II, defined by at least one major depressive episode and one hypomanic episode (Pan et al., 2024; Singh et al., 2025). BD affects approximately 40 million people worldwide (World Health Organization, 2024; Singh et al., 2025; Institute for Health Metrics and Evaluation, 2024). At the neurobiological level, BD is associated with widespread structural and functional brain alterations, including cortical thinning and white matter abnormalities that worsen with recurrent episodes (Singh et al., 2025; Bai et al., 2020). Functional studies reveal dysregulation of frontolimbic circuits, with altered limbic reactivity and reduced prefrontal engagement during emotional and cognitive processing (Singh et al., 2025). In general, BD is increasingly conceptualized as a multifactorial disorder involving genetic vulnerability, mitochondrial dysfunction, inflammation, and metabolic dysregulation (Singh et al., 2025; Khayachi et al., 2025). Mitochondrial abnormalities, including impaired energy production, oxidative stress, and altered electron transport chain activity, are consistently reported, together with mitochondrial DNA changes (Singh et al., 2025; Khayachi et al., 2025; Lam et al., 2023). In parallel, disrupted insulin signaling and altered cerebral glucose metabolism support the concept of BD as a disorder of energy homeostasis, with metabolic fluctuations across mood states (Bai et al., 2020; Khayachi et al., 2025). Inflammatory alterations are also observed even during euthymia and may contribute to microglial activation, excitotoxicity, and circuit dysfunction (Bai et al., 2020; Khayachi et al., 2025).

In this context, evidence linking miR-7 to BD remains limited and mainly associative. Most studies have focused on peripheral biomarkers rather than mechanistic insight. Lee et al. (2020) reported increased serum levels of miR-7-5p in BD-II patients, suggesting its potential as a circulating biomarker. The same group later observed a positive correlation between miR-7-5p and plasma BDNF levels in BD-II, although this association contrasts with reports of BDNF repression by miR-7-5p in other contexts and cannot establish causality (Li et al., 2019). More recently, Tsai et al. (2024) analyzed phenylalanyl-tRNA synthetase beta subunit (FARSB), a protein involved in translational regulation, and found that its levels correlate positively with BDNF in BD-II patients. They proposed that miR-7-5p may interact with FARSB and indirectly modulate BDNF signaling (Tsai et al., 2024), although this mechanism remains speculative and requires experimental validation. Therefore, miR-7 emerges as a potential but unconfirmed regulator in BD. While mitochondrial dysfunction, inflammation, and metabolic dysregulation, core features of BD, could theoretically involve miR-7-related pathways, direct mechanistic evidence is still lacking. Likewise, key targets such as BDNF and synaptic genes including *SHANK3* remain to be functionally explored.

### 4.3 Other neuropsychiatric disorders

Beyond SCZ and BD, miR-7 has also been detected in hippocampal histological samples from patients with major depressive disorder (MDD), although its signal appears relatively low compared to other miRNAs (Søkilde et al., 2025). In addition, no significant changes in circulating levels have been reported in blood samples (Liu X. et al., 2024). The heterogeneity and spectrum-like nature of psychiatric disorders, together with the lack of direct studies addressing the role of miR-7 across different conditions, currently preclude drawing firm conclusions. Nevertheless, expanding research on miR-7 to other disorders, such as MDD or autism, could be of particular interest, given the involvement of metabolic alterations like mitochondrial dysfunction and redox imbalance, in the pathophysiology of these conditions (Kim et al., 2019).

## 5 Brain cancer

### 5.1 Glioblastoma multiforme

GBM is the most common malignant brain tumor, accounting for over 50% of high-grade gliomas, with a global incidence of 3–5 cases per 100,000 people per year (Torrecilla-Parra et al., 2025; Martínez-Garcia et al., 2018; Sipos et al., 2025). Most cases arise *de novo*, while secondary GBMs develop from lower-grade gliomas (Martínez-Garcia et al., 2018). GBM is defined as a grade IV tumor with the poorest prognosis among infiltrating gliomas (Sipos et al., 2025; Louis et al., 2016). Common manifestations of GBM include persistent headaches, seizures, and cognitive or behavioural changes such as memory impairment, changes in personality, and disorientation. Patients may also develop localised neurological impairments, including unilateral weakness or sensory loss, visual and language problems (Sipos et al., 2025). At the cellular level tumor metabolic switching is a hallmark of GBM, characterised by the preferential use of aerobic glycolysis, also known as the Warburg effect, which increases glucose uptake and lactate production in the presence of oxygen, compensating for insufficient oxidative phosphorylation (Pavlova et al., 2022; Guntuku et al., 2016). It is partly driven by the aberrant expression of oncogenes and tumor suppressor genes. This leads to enhanced biomass production and lactate-mediated acidification of the tumor microenvironment, thereby facilitating invasion and the aggressive progression of GBM (Liberti and Locasale, 2016; Guntuku et al., 2016). Aerobic glycolysis in GBM is regulated by glucose transporters (GLUT1–4) and glycolytic enzymes, and is further influenced by key signalling pathways, including PI3K/AKT, p53, epidermal growth factor receptor (EGFR), etc. (Guntuku et al., 2016). Additionally, mitochondrial dysfunction and morphological abnormalities in GBM compromise OXPHOS-mediated ATP production and reduce apoptosis. Mutations in isocitrate dehydrogenases 1 and 2 (IDH1 and IDH2), key NADPH-linked mitochondrial enzymes in the TCA cycle, impair mitochondrial energy production, providing clear evidence of metabolic dysfunction in gliomas (Guntuku et al., 2016). Treatment for GBM typically involves surgical resection, radiotherapy, and temozolomide (TMZ) chemotherapy. However, it remains largely ineffective due to incomplete tumor resection and intrinsic treatment resistance (Torrecilla-Parra et al., 2025; Singh et al., 2021). In this context, miRNAs have emerged as promising therapeutic tools to target tumor metabolism (Ordóñez-Rubiano et al., 2024).

MiR-7 is abnormally downregulated in GBM and has been linked with the inhibition of EGFR and its downstream signaling cascades, including the PI3K/AKT and RAF/MEK/ERK pathways, which are essential for tumor survival and proliferation (Kefas et al., 2008). Consistently, miR-7-5p overexpression has demonstrated to impair GBM cell growth by inducing cell cycle arrest through the coordinated inhibition of these pathways, highlighting its broader tumor-suppressive function (Wang et al., 2023; Liu et al., 2014). In addition, miR-7 regulates GBM cell migration and invasion by suppressing, among other targets, focal adhesion kinase (FAK), special AT-rich sequence-binding protein-1 (STATB1), and ERK/AKT signaling pathways (Torrecilla-Parra et al., 2025; Liu et al., 2014; Wu et al., 2011; Shukla et al., 2018; Yin et al., 2019; Pan et al., 2020). In this line, miR-7 also contributes to tumor resistance by regulating cancer stem cell properties and therapy response (Jia et al., 2019), further reinforcing its anti-tumoral role. Extending its tumor-suppressive effects, Torrecilla-Parra et al. (2025) demonstrated that miR-7 overexpression also disrupts cellular bioenergetics in GBM by simultaneously impairing glycolysis and mitochondrial function. Specifically, miR-7 suppresses glycolytic flux through posttranscriptional repression of key target enzymes such as neuronal enolase (ENO2). This inhibition reduces glycolytic capacity and limits pyruvate production, thereby restricting substrate availability for the TCA cycle and ultimately compromising oxidative phosphorylation. Consequently, cells experience a severe energetic deficit characterized by reduced ATP production and impaired mitochondrial respiratory capacity. This bioenergetic collapse is accompanied by increased oxidative stress and marked alterations in mitochondrial morphology, reflecting a failure in mitochondrial quality control (Torrecilla-Parra et al., 2025). Additionally, miR-7 has been shown to modulate the autophagic process (Torrecilla-Parra et al., 2025; Chakrabarti and Ray, 2016) via dual-stage modulation that promotes its initiation through inhibition of the PI3K/AKT/mTOR pathway, while simultaneously blocking autophagic flux at later stages through posttranscriptional repression of key SNARE proteins, STX17 and SNAP29, required for autolysosome formation (Torrecilla-Parra et al., 2025). Furthermore, Torrecilla-Parra et al. identified additional miR-7 targets involved in lysosomal biology, including BLOC1S4 and SCARB2, both critical for vesicular trafficking and lysosomal function (Torrecilla-Parra et al., 2025; Rudnik et al., 2024; Dell'Angelica, 2004; Reczek et al., 2007). These multifaceted function on metabolic dysregulation in GBM were reflected *in vivo*, where miR-7 overexpression in tumor xenograft led to a striking reduction in tumor size and volume, by interfering in these cellular processes (Torrecilla-Parra et al., 2025). Altogether, these findings position miR-7 as a potential metabomiR in GBM, integrating the crosstalk between metabolic and proliferative programs with relevant therapeutic implications. Interestingly, these findings may also provide insight into neurodegenerative disorders. In AD, where miR-7 is consistently upregulated and impaired autophagic flux is a well-recognized pathological feature, persistent miR-7 overexpression could potentially exacerbate defects in autophagosome maturation and lysosomal function. Although this hypothesis remains to be experimentally validated in neurodegenerative diseases, it further supports the concept that the biological consequences of miR-7-mediated autophagy are highly context-dependent. Future studies should address its role in metabolically plastic populations, particularly glioma stem cells, which couple high proliferative capacity with adaptive metabolic states and drive tumor recurrence and therapeutic resistance.

### 5.2 Brain metastasis

Brain metastasis refers to the spread of cancer cells from a primary tumor outside the CNS to the brain, where they disseminate through the bloodstream or lymphatic system and establish secondary tumors that retain a similar phenotype to the primary lesion (Bergers and Fendt, 2021). Metastatic lesions represent the most prevalent form of malignant tumors affecting the CNS. It is estimated that between 20 and 40% of individuals with cancer will develop brain involvement at some point during disease progression. The tumors most commonly responsible for brain dissemination originate from the lung (≥50%), followed by breast cancer (15–25%) and melanoma (5–20%), although virtually any malignancy has the potential to spread to the CNS (Eichler and Loeffler, 2007; Bertolini et al., 2015). The clinical manifestations of brain metastases largely overlap with those observed in primary brain tumors, including headaches, seizures, and focal neurological impairments which may lead to altered consciousness and reduced cognitive function (Bertolini et al., 2015; Schreurs et al., 2025).

The brain represents a highly specialized metastatic niche composed of neurons, glial cells, endothelial cells, pericytes, and a distinct ECM, within an active neuroimmune environment (Bertolini et al., 2015). Although once considered immune-privileged due to the blood–brain barrier, it is now recognized that tumor–host interactions critically shape metastatic colonization and progression. In this context, cancer stem cells (CSCs) represent a slow-cycling, self-renewing population that contributes to tumor maintenance and long-term persistence within the brain niche (Okuda et al., 2013). In parallel, metastatic cancer cells undergo metabolic reprogramming driven by both tumor origin and brain-specific constraints. In brain metastases, alterations in oxidative metabolism are frequently observed, and alternative substrates such as acetate can sustain energy production and tumor survival, as reported in metastases from lung and breast cancers (Bergers and Fendt, 2021).

As mentioned earlier, studies across different cancer models have identified miR-7-5p as an important regulator of tumor biology. Increasing evidence suggests a context-specific role of miR-7 in brain metastasis, where it contributes to tumor adaptation within the neural microenvironment (Okuda et al., 2013; Mirzaei et al., 2024). In lung cancer brain metastases, miR-7 is downregulated, promoting proliferation, epithelial–mesenchymal transition, and migration, and has been proposed as a biomarker capable of distinguishing primary from metastatic brain tumors of pulmonary origin (Nikolova et al., 2022). Similarly, in breast cancer, miR-7 is reduced in cancer stem cells derived from brain and bone metastases, where it regulates self-renewal capacity. Mechanistically, miR-7 targets key oncogenic drivers such as EGFR and IRS-1, and suppresses stemness-associated factors including KLF4, whose expression inversely correlates with miR-7 levels. Restoring miR-7 expression selectively impairs brain metastatic growth, with limited effects on bone metastasis, highlighting a brain-specific functional role (Okuda et al., 2013).

In GBM, additional targets such as TBX2 further link miR-7 to epithelial–mesenchymal transition and invasive behavior, reinforcing its role in processes essential for tumor dissemination within the brain (Pan et al., 2020).

In summary, metabolic reprogramming, together with inhibition of proliferation, epithelial–mesenchymal transition, migration, and ECM degradation, underlies the tumor-suppressive effects associated with miR-7 restoration in brain metastasis. These observations support miR-7 as a promising therapeutic strategy for limiting metastatic dissemination. However, its context-dependent mechanisms across distinct brain metastatic niches remain to be fully elucidated.

## 6 Limitations and considerations of the available evidence

**Figure 3:**
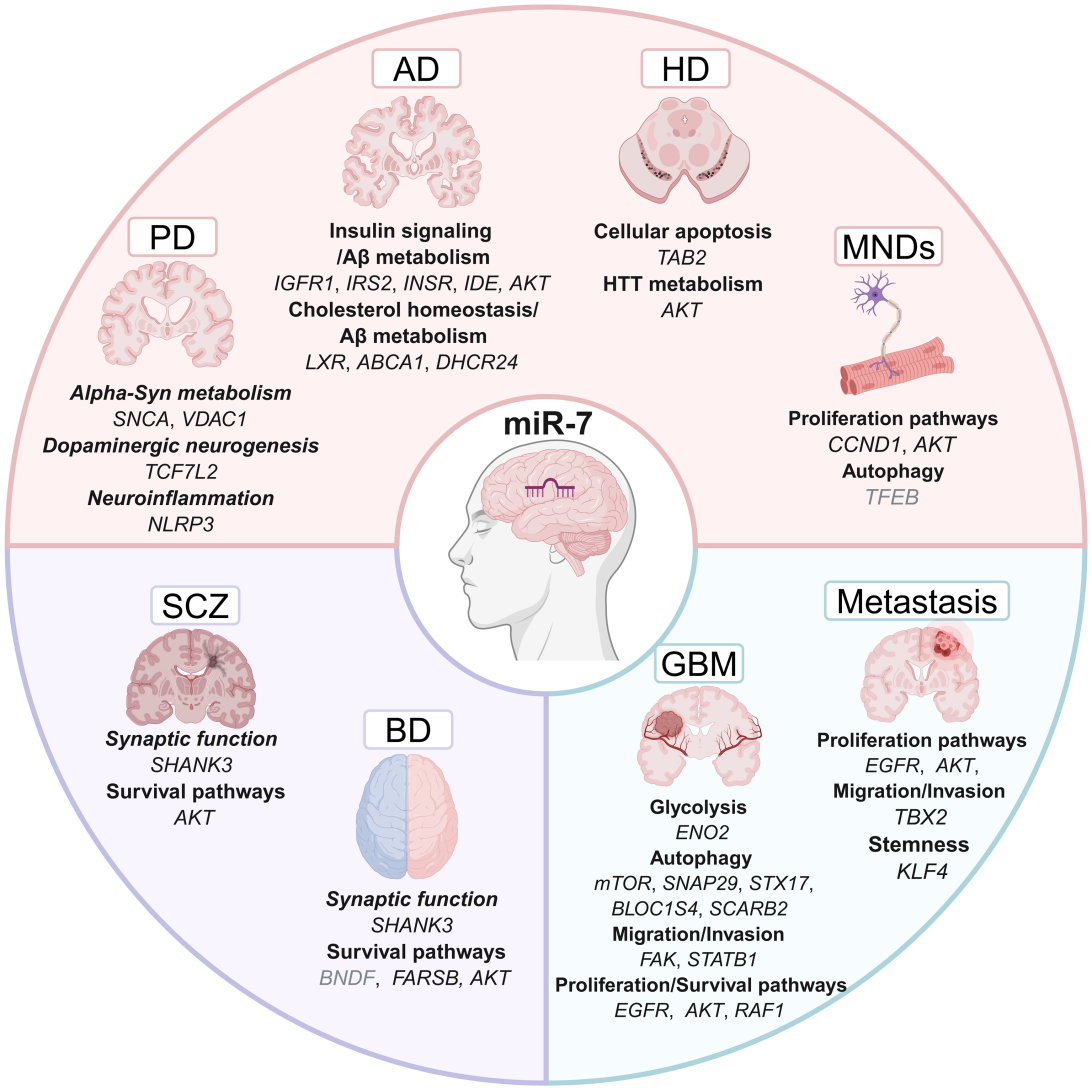
Schematic overview of the role of miR-7 in the metabolic regulation of brain disorders. Schematic representation of miR-7-associated cellular pathways across major neurological diseases, highlighting both regulated processes and associated genes. Genes highlighted in grey are linked to miR-7 activity, whereas genes in black represent direct miR-7 targets involved in the metabolic processes shown in bold, which are central to each disease category: Neurodegenerative disorders (PD, Parkinson’s disease; AD, Alzheimer’s disease; HD, Huntington’s disease; MNDs, motor neuron diseases), psychiatric disorders (SCZ, schizophrenia; BD, bipolar disorder), and brain cancer (GBM, glioblastoma multiforme; BM, brain metastasis; Aβ, Amyloid-β, HTT, Huntingtin). Created with BioRender.com.

This review summarizes the emerging role of miR-7 in metabolic regulation across neurological disorders (Figure 3). Overall, the strength of the available evidence varies considerably across the pathological conditions discussed. The most robust mechanistic evidence currently supports a role for miR-7 in Alzheimer’s disease, largely owing to its well-established involvement in Aβ metabolism, in Parkinson’s disease through the regulation of *α*-syn accumulation and mitochondrial homeostasis, and in glioblastoma, where extensive experimental evidence has demonstrated its tumor-suppressive role by targeting multiple oncogenic and metabolic pathways. In contrast, evidence for Huntington’s disease, ALS, schizophrenia, bipolar disorder and brain metastasis remains comparatively more limited, being supported by fewer mechanistic studies or predominantly associative findings. In neurodegenerative diseases in particular, dysregulated miR-7 levels coincide with alterations in multiple metabolic pathways, where its molecular targets and mechanisms are better characterized and the link between metabolism and neurodegeneration is more clearly established.

Notably, these alterations are often exacerbated by ageing, a major risk factor for both neurodegenerative diseases and cancer, characterized by reduced metabolic flexibility and impaired glucose and lipid homeostasis (López-Otín et al., 2013). However, most studies to date have focused predominantly on glucose metabolism, with lipid biology remaining comparatively underexplored despite increasing evidence linking lipid homeostasis to neuronal function, inflammation, and tumor progression. In this context, miR-7 emerges as a potential upstream regulator of lipid homeostasis in the brain, suggesting a mechanistic link between its dysregulation and metabolic vulnerability across neurological and neuro-oncological diseases.

Although the role of miR-7 in neuroscience remains relatively underexplored, its high expression in the brain, its modulation during neurodevelopment, and its differential expression under pathological conditions have positioned it as a promising candidate for novel therapeutic strategies. Within this context of metabolic imbalance, miR-7 appears to play a context-dependent role. In conditions such as PD, SCZ, and brain cancer, it has been associated with neuroprotective functions, including the regulation of neuroinflammation, protein homeostasis, and cellular metabolism. In contrast, in other contexts such as AD, its role may differ, reflecting the complexity of its regulatory network. These differences likely arise from the broad range of processes controlled by miR-7, which extend beyond metabolism to include cell proliferation, intracellular signaling, ECM dynamics, and immune responses. Moreover, many of the findings regarding the role of this miRNA in disease derive from clinical studies, where variability between patient cohorts should be carefully considered when interpreting results.

Further adding complexity, miRNAs do not act in isolation. Several studies have shown that miR-7 functions correlate with other miRNAs, such as miR-223 or miR-153 in PD (Fragkouli and Doxakis, 2014; Citterio et al., 2023) or miR-504 in neuropsychiatric disorders (Choi et al., 2015). Moreover, miR-7 function may depend on complex regulatory networks, including interactions with RNA binding proteins or long non-coding RNA. Importantly, the specific genomic location of miR-7-1 and miR-7-3, within hnRNPK and miR-7-3HG, respectively, represents an unusual arrangement in nature which may have functional biological significance This suggests the existence of interconnected feedback loops that may fine-tune miR-7 activity, which remain largely unexplored in the context of brain diseases (Choudhury et al., 2013).

Finally, it is important to highlight that miRNAs hold considerable therapeutic potential, although their successful clinical translation requires overcoming several key challenges. These include limited cellular uptake due to their hydrophilic and negatively charged nature, restricted membrane permeability, and susceptibility to degradation following endocytosis, as well as potential off-target and immune-related effects upon systemic administration (Roberts et al., 2020). To address these limitations, a variety of delivery strategies are being actively developed, including viral and non-viral vectors, nanoparticle-based systems, and extracellular vesicles, which can improve stability, cellular uptake, and tissue specificity (Lundstrom, 2020; Liu and Berkhout, 2011). Notably, emerging platforms such as graphene oxide nanosheets have shown promise as efficient *in vivo* delivery vehicles for miR-7-based therapies (Kutwin et al., 2024). Continued optimization of these approaches will be essential to fully harness the therapeutic potential of miRNAs in clinical settings.

## 7 Concluding remarks

In summary, these findings position miR-7 as a relevant integrator of key metabolic and cellular pathways across neurological diseases. Nevertheless, its effects depend on the pathological context, as it may exert either neuroprotective or detrimental roles depending on the disease. Accordingly, its context-specific mechanisms of action remain to be fully elucidated. Future studies integrating molecular, metabolic, and clinical data will be essential to better define its functional relevance and to translate these insights into effective therapeutic strategies.

## References

[ref1] Adusumilli L. Facchinello N. Teh C. Busolin G. Le M. T. Yang H. . (2020). *miR-7* controls the dopaminergic/Oligodendroglial fate through Wnt/β-catenin signaling regulation. Cells 9:711. doi: 10.3390/cells9030711, 32183236 PMC7140713

[ref2] Agbu P. Carthew R. W. (2021). MicroRNA-mediated regulation of glucose and lipid metabolism. Nat. Rev. Mol. Cell Biol. 22, 425–438. doi: 10.1038/s41580-021-00354-w, 33772227 PMC8853826

[ref3] Amar L. Benoit C. Beaumont G. Vacher C. M. Crepin D. Taouis M. . (2012). MicroRNA expression profiling of hypothalamic arcuate and paraventricular nuclei from single rats using Illumina sequencing technology. J. Neurosci. Methods 209, 134–143. doi: 10.1016/j.jneumeth.2012.05.033, 22687940

[ref4] Ambros V. (2004). The functions of animal microRNAs. Nature 431, 350–355. doi: 10.1038/nature02871, 15372042

[ref5] Ávila-Villanueva M. Marcos Dolado A. Gómez-Ramírez J. Fernández-Blázquez M. (2022). Brain structural and functional changes in cognitive impairment due to Alzheimer's disease. Front. Psychol. 13:886619. doi: 10.3389/fpsyg.2022.886619, 35800946 PMC9253821

[ref6] Bai Y. M. Chen M. H. Hsu J. W. Huang K. L. Tu P. C. Chang W. C. . (2020). A comparison study of metabolic profiles, immunity, and brain gray matter volumes between patients with bipolar disorder and depressive disorder. J. Neuroinflammation 17:42. doi: 10.1186/s12974-020-1724-9, 32000805 PMC6990475

[ref7] Bai X. Mai M. Yao K. Zhang M. Huang Y. Zhang W. . (2022). The role of DHCR24 in the pathogenesis of AD: re-cognition of the relationship between cholesterol and AD pathogenesis. Acta Neuropathol. Commun. 10:35. doi: 10.1186/s40478-022-01338-3, 35296367 PMC8925223

[ref8] Barone C. Qi X. (2023). Altered metabolism in motor neuron diseases: mechanism and potential therapeutic target. Cells 12:1536. doi: 10.3390/cells12111536, 37296656 PMC10252517

[ref9] Bergers G. Fendt S. M. (2021). The metabolism of cancer cells during metastasis. Nat. Rev. Cancer 21, 162–180. doi: 10.1038/s41568-020-00320-2, 33462499 PMC8733955

[ref10] Bertolini F. Spallanzani A. Fontana A. Depenni R. Luppi G. (2015). Brain metastases: an overview. CNS Oncol. 4, 37–46. doi: 10.2217/cns.14.51, 25586424 PMC6093020

[ref11] Beveridge N. J. Gardiner E. Carroll A. P. Tooney P. A. Cairns M. J. (2010). Schizophrenia is associated with an increase in cortical microRNA biogenesis. Mol. Psychiatry 15, 1176–1189. doi: 10.1038/mp.2009.84, 19721432 PMC2990188

[ref12] Braak H. Del Tredici K. (2011). The pathological process underlying Alzheimer's disease in individuals under thirty. Acta Neuropathol. 121, 171–181. doi: 10.1007/s00401-010-0789-4, 21170538

[ref13] Brisch R. Saniotis A. Wolf R. Bielau H. Bernstein H. G. Steiner J. . (2014). The role of dopamine in schizophrenia from a neurobiological and evolutionary perspective: old fashioned, but still in vogue. Front. Psych. 5:47. doi: 10.3389/fpsyt.2014.00047, 24904434 PMC4032934

[ref14] Bushati N. Cohen S. M. (2008). MicroRNAs in neurodegeneration. Curr. Opin. Neurobiol. 18, 292–296. doi: 10.1016/j.conb.2008.07.001, 18662781

[ref15] Cao D. D. Li L. Chan W. Y. (2016). MicroRNAs: key regulators in the central nervous system and their implication in neurological diseases. Int. J. Mol. Sci. 17:842. doi: 10.3390/ijms170608421 27240359 PMC4926376

[ref16] Cao B. Wang T. Qu Q. Kang T. Yang Q. (2018). Long noncoding RNA SNHG1 promotes Neuroinflammation in Parkinson's disease via regulating miR-7/NLRP3 pathway. Neuroscience 388, 118–127. doi: 10.1016/j.neuroscience.2018.07.019, 30031125

[ref17] Chakrabarti M. Ray S. K. (2016). Anti-tumor activities of luteolin and silibinin in glioblastoma cells: overexpression of miR-7-1-3p augmented luteolin and silibinin to inhibit autophagy and induce apoptosis in glioblastoma in vivo. Apoptosis 21, 312–328. doi: 10.1007/s10495-015-1198-x, 26573275

[ref18] Chaudhuri A. D. Choi D. C. Kabaria S. Tran A. Junn E. (2016). MicroRNA-7 regulates the function of mitochondrial permeability transition pore by targeting VDAC1 expression. J. Biol. Chem. 291, 6483–6493. doi: 10.1074/jbc.M115.691352, 26801612 PMC4813563

[ref19] Chen Y. Guan Y. Liu H. Wu X. Yu L. Wang S. . (2012). Activation of the Wnt/β-catenin signaling pathway is associated with glial proliferation in the adult spinal cord of ALS transgenic mice. Biochem. Biophys. Res. Commun. 420, 397–403. doi: 10.1016/j.bbrc.2012.03.006, 22426476

[ref20] Chen C. Guo M. Zhao X. Zhao J. Chen L. He Z. . (2023). MicroRNA-7: a new intervention target for inflammation and related diseases. Biomolecules 13:1185. doi: 10.3390/biom13081185, 37627250 PMC10452300

[ref21] Chen Z. S. Peng S. I. Leong L. I. Gall-Duncan T. Wong N. S. J. Li T. H. . (2025). Mutant huntingtin induces neuronal apoptosis via derepressing the non-canonical poly(a) polymerase PAPD5. Nat. Commun. 16:3307. doi: 10.1038/s41467-025-58618-41 40204699 PMC11982267

[ref22] Choi S. Y. Pang K. Kim J. Y. Ryu J. R. Kang H. Liu Z. . (2015). Post-transcriptional regulation of SHANK3 expression by microRNAs related to multiple neuropsychiatric disorders. Mol. Brain 8:74. doi: 10.1186/s13041-015-0165-3, 26572867 PMC4647645

[ref23] Choudhury N. R. de Lima Alves F. de Andrés-Aguayo L. Graf T. Cáceres J. F. Rappsilber J. . (2013). Tissue-specific control of brain-enriched miR-7 biogenesis. Genes Dev. 27, 24–38. doi: 10.1101/gad.199190.112, 23307866 PMC3553281

[ref24] Christodoulou R. C. Eller D. Papageorgiou P. S. Angelopoulou E. Vassiliou E. Papageorgiou S. G. (2026). Metabolic dysfunction in Alzheimer's disease: brain glucose Hypometabolism as an early precursor to amyloid and tau pathology. J. Clin. Med. 15:1884. doi: 10.3390/jcm15051884, 41827301 PMC12986246

[ref25] Citterio L. A. Mancuso R. Agostini S. Meloni M. Clerici M. (2023). Serum and Exosomal miR-7-1-5p and miR-223-3p as possible biomarkers for Parkinson's disease. Biomolecules 13:865. doi: 10.3390/biom13050865, 37238734 PMC10216839

[ref26] Claborn M. K. Stevens D. L. Walker C. K. Gildon B. L. (2019). Nusinersen: a treatment for spinal muscular atrophy. Ann. Pharmacother. 53, 61–69. doi: 10.1177/1060028018789956, 30008228

[ref27] Codolo G. Plotegher N. Pozzobon T. Brucale M. Tessari I. Bubacco L. . (2013). Triggering of inflammasome by aggregated α-synuclein, an inflammatory response in synucleinopathies. PLoS One 8:e55375. doi: 10.1371/journal.pone.0055375, 23383169 PMC3561263

[ref28] Colin E. Régulier E. Perrin V. Dürr A. Brice A. Aebischer P. . (2005). Akt is altered in an animal model of Huntington's disease and in patients. Eur. J. Neurosci. 21, 1478–1488. doi: 10.1111/j.1460-9568.2005.03985.x, 15845076

[ref29] Cortes C. J. Miranda H. C. Frankowski H. Batlevi Y. Young J. E. Le A. . (2014). Polyglutamine-expanded androgen receptor interferes with TFEB to elicit autophagy defects in SBMA. Nat. Neurosci. 17, 1180–1189. doi: 10.1038/nn.3787, 25108912 PMC4180729

[ref30] Dai M. H. Zheng H. Zeng L. D. Zhang Y. (2017). The genes associated with early-onset Alzheimer's disease. Oncotarget 9, 15132–15143. doi: 10.18632/oncotarget.23738, 29599933 PMC5871104

[ref31] Dell'Angelica E. C. (2004). The building BLOC(k)s of lysosomes and related organelles. Curr. Opin. Cell Biol. 16, 458–464. doi: 10.1016/j.ceb.2004.05.001, 15261680

[ref32] Devrome M. Casteels C. Van der Perren A. Van Laere K. Baekelandt V. Koole M. (2019). Identifying a glucose metabolic brain pattern in an adeno-associated viral vector based rat model for Parkinson's disease using 18F-FDG PET imaging. Sci. Rep. 9:12368. doi: 10.1038/s41598-019-48713-0, 31451742 PMC6710432

[ref33] Dong X. Cong S. (2021). MicroRNAs in Huntington's disease: diagnostic biomarkers or therapeutic agents? Front. Cell. Neurosci. 15:705348. doi: 10.3389/fncel.2021.705348, 34421543 PMC8377808

[ref34] Dong-Chen X. Yong C. Yang X. Chen-Yu S. Li-Hua P. (2023). Signaling pathways in Parkinson's disease: molecular mechanisms and therapeutic interventions. Signal Transduct. Target. Ther. 8:73. doi: 10.1038/s41392-023-01353-3, 36810524 PMC9944326

[ref35] Doxakis E. (2010). Post-transcriptional regulation of alpha-synuclein expression by miR-7 and miR-153. J. Biol. Chem. 285, 12726–12734. doi: 10.1074/jbc.M109.086827, 20106983 PMC2857101

[ref36] D'Silva A. M. Kariyawasam D. Venkat P. Mayoh C. Farrar M. A. (2023). Identification of novel CSF-derived miRNAs in treated paediatric onset spinal muscular atrophy: an exploratory study. Pharmaceutics 15:170. doi: 10.3390/pharmaceutics15010170, 36678797 PMC9865256

[ref37] Dupuis L. Pradat P. F. Ludolph A. C. Loeffler J. P. (2011). Energy metabolism in amyotrophic lateral sclerosis. Lancet Neurol. 10, 75–82. doi: 10.1016/S1474-4422(10)70224-6, 21035400

[ref38] Edens B. M. Ajroud-Driss S. Ma L. Ma Y. C. (2015). Molecular mechanisms and animal models of spinal muscular atrophy. Biochim. Biophys. Acta 1852, 685–692. doi: 10.1016/j.bbadis.2014.07.024, 25088406 PMC12184990

[ref39] Eichler A. F. Loeffler J. S. (2007). Multidisciplinary management of brain metastases. Oncologist 12, 884–898. doi: 10.1634/theoncologist.12-7-884, 17673619

[ref40] Emamian E. S. (2012). AKT/GSK3 signaling pathway and schizophrenia. Front. Mol. Neurosci. 5:33. doi: 10.3389/fnmol.2012.00033, 22435049 PMC3304298

[ref41] Emanuele M. Esposito A. Camerini S. Antonucci F. Ferrara S. Seghezza S. . (2016). Exogenous alpha-Synuclein alters pre- and post-synaptic activity by fragmenting lipid rafts. EBioMedicine 7, 191–204. doi: 10.1016/j.ebiom.2016.03.038, 27322472 PMC4909369

[ref42] Evans C. S. Holzbaur E. L. F. (2019). Autophagy and mitophagy in ALS. Neurobiol. Dis. 122, 35–40. doi: 10.1016/j.nbd.2018.07.005, 29981842 PMC6366665

[ref43] Fan Z. Lu M. Qiao C. Zhou Y. Ding J. H. Hu G. (2016). MicroRNA-7 enhances subventricular zone neurogenesis by inhibiting NLRP3/Caspase-1 Axis in adult neural stem cells. Mol. Neurobiol. 53, 7057–7069. doi: 10.1007/s12035-015-9620-5, 26676570

[ref44] Fayemendy P. Marin B. Labrunie A. Boirie Y. Walrand S. Achamrah N. . (2021). Hypermetabolism is a reality in amyotrophic lateral sclerosis compared to healthy subjects. J. Neurol. Sci. 420:117257. doi: 10.1016/j.jns.2020.117257, 33290920

[ref45] Feldman E. L. Goutman S. A. Petri S. Mazzini L. Savelieff M. G. Shaw P. J. . (2022). Amyotrophic lateral sclerosis. Lancet 400, 1363–1380. doi: 10.1016/S0140-6736(22)01272-7, 36116464 PMC10089700

[ref46] Fernández-Castillo N. Martín-García E. (2022). Editorial: genetic and epigenetic mechanisms underpinning vulnerability to developing psychiatric disorders. Front. Psych. 13:917198. doi: 10.3389/fpsyt.2022.917198, 35770055 PMC9234658

[ref47] Fernández-de Frutos M. Galán-Chilet I. Goedeke L. Kim B. Pardo-Marqués V. Pérez-García A. . (2019). MicroRNA 7 impairs insulin signaling and regulates aβ levels through posttranscriptional regulation of the insulin receptor substrate 2, insulin receptor, insulin-degrading enzyme, and liver X receptor pathway. Mol. Cell. Biol. 39:e00170-19. doi: 10.1128/MCB.00170-19, 31501273 PMC6817752

[ref48] Firbank M. J. Yarnall A. J. Lawson R. A. Duncan G. W. Khoo T. K. Petrides G. S. . (2017). Cerebral glucose metabolism and cognition in newly diagnosed Parkinson's disease: ICICLE-PD study. J. Neurol. Neurosurg. Psychiatry 88, 310–316. doi: 10.1136/jnnp-2016-313918, 28315844

[ref49] Fortanier E. Grapperon A. M. Le Troter A. Verschueren A. Ridley B. Guye M. . (2019). Structural connectivity alterations in amyotrophic lateral sclerosis: a graph theory based imaging study. Front. Neurosci. 13:1044. doi: 10.3389/fnins.2019.01044, 31632235 PMC6783612

[ref50] Fortin D. L. Troyer M. D. Nakamura K. Kubo S. Anthony M. D. Edwards R. H. (2004). Lipid rafts mediate the synaptic localization of alpha-synuclein. J. Neurosci. 24, 6715–6723. doi: 10.1523/JNEUROSCI.1594-04.2004, 15282274 PMC6729723

[ref51] Fragkouli A. Doxakis E. (2014). miR-7 and miR-153 protect neurons against MPP(+)-induced cell death via upregulation of mTOR pathway. Front. Cell. Neurosci. 8:182. doi: 10.3389/fncel.2014.00182, 25071443 PMC4080263

[ref52] Frutos M. F. Pardo-Marqués V. Torrecilla-Parra M. Rada P. Pérez-García A. Martín-Martín Y. . (2023). MiR-7 controls cholesterol biosynthesis through posttranscriptional regulation of DHCR24 expression. Biochimic*a Biophy. Acta* 1866:194938. doi: 10.1016/j.bbagrm.2023.194938, 37086967

[ref53] Gao Y. Li J. Zhang Z. Zhang R. Pollock A. Sun T. (2019). MicroRNA miR-7 and miR-17-92 in the arcuate nucleus of mouse hypothalamus regulate sex-specific diet-induced obesity. Mol. Neurobiol. 56, 7508–7521. doi: 10.1007/s12035-019-1618-yCC 31044367

[ref54] Ghafouri-Fard S. Eghtedarian R. Taheri M. Beatrix Brühl A. Sadeghi-Bahmani D. Brand S. (2021). A review on the expression pattern of non-coding RNAs in patients with schizophrenia: with a special focus on peripheral blood as a source of expression analysis. Front. Psych. 12:640463. doi: 10.3389/fpsyt.2021.640463, 34220567 PMC8249727

[ref55] Goedeke L. Fernández-Hernando C. (2012). Regulation of cholesterol homeostasis. Cell. Mol. Life Sci. 69, 915–930. doi: 10.1007/s00018-011-0857-5, 22009455 PMC11114919

[ref56] Gomes B. C. Peixinho N. Pisco R. Gromicho M. Pronto-Laborinho A. C. Rueff J. . (2023). Differential expression of miRNAs in amyotrophic lateral sclerosis patients. Mol. Neurobiol. 60, 7104–7117. doi: 10.1007/s12035-023-03520-7, 37531027 PMC10657797

[ref57] Gordon R. Albornoz E. A. Christie D. C. Langley M. R. Kumar V. Mantovani S. . (2018). Inflammasome inhibition prevents α-synuclein pathology and dopaminergic neurodegeneration in mice. Sci. Transl. Med. 10:eaah4066. doi: 10.1126/scitranslmed.aah4066 30381407 PMC6483075

[ref58] Guntuku L. Naidu V. G. Yerra V. G. (2016). Mitochondrial dysfunction in gliomas: Pharmacotherapeutic potential of natural compounds. Curr. Neuropharmacol. 14, 567–583. doi: 10.2174/1570159x14666160121115641, 26791479 PMC4981742

[ref59] Gustavsson A. Norton N. Fast T. Frölich L. Georges J. Holzapfel D. . (2023). Global estimates on the number of persons across the Alzheimer's disease continuum. Alzheimers Dement. 19, 658–670. doi: 10.1002/alz.12694, 35652476

[ref60] Gwathmey K. G. Corcia P. McDermott C. J. Genge A. Sennfält S. de Carvalho M. . (2023). Diagnostic delay in amyotrophic lateral sclerosis. Eur. J. Neurol. 30, 2595–2601. doi: 10.1111/ene.15874, 37209406

[ref61] Hansen T. B. Jensen T. I. Clausen B. H. Bramsen J. B. Finsen B. Damgaard C. K. . (2013). Natural RNA circles function as efficient microRNA sponges. Nature 495, 384–388. doi: 10.1038/nature11993, 23446346

[ref62] Hawley Z. C. E. Campos-Melo D. Droppelmann C. A. Strong M. J. (2017). MotomiRs: miRNAs in motor neuron function and disease. Front. Mol. Neurosci. 10:127. doi: 10.3389/fnmol.2017.00127, 28522960 PMC5415563

[ref63] Heni M. (2024). The insulin resistant brain: impact on whole-body metabolism and body fat distribution. Diabetologia 67, 1181–1191. doi: 10.1007/s00125-024-06104-9, 38363340 PMC11153284

[ref64] Herzer S. Silahtaroglu A. Meister B. (2012). Locked nucleic acid-based in situ hybridisation reveals miR-7a as a hypothalamus-enriched microRNA with a distinct expression pattern. J. Neuroendocrinol. 24, 1492–1504. doi: 10.1111/j.1365-2826.2012.02358.x, 22775435

[ref65] Horsham J. L. Kalinowski F. C. Epis M. R. Ganda C. Brown R. A. Leedman P. J. (2015). Clinical potential of microRNA-7 in Cancer. J. Clin. Med. 4, 1668–1687. doi: 10.3390/jcm4091668, 26308064 PMC4600152

[ref66] Hu W. MacDonald M. L. Elswick D. E. Sweet R. A. (2015). The glutamate hypothesis of schizophrenia: evidence from human brain tissue studies. Ann. N. Y. Acad. Sci. 1338, 38–57. doi: 10.1111/nyas.12547, 25315318 PMC4363164

[ref67] Huang C. Voglewede M. M. Ozsen E. N. Wang H. Zhang H. (2023). SHANK3 mutations associated with autism and schizophrenia Lead to shared and distinct changes in dendritic spine dynamics in the developing mouse brain. Neuroscience 528, 1–11. doi: 10.1016/j.neuroscience.2023.07.024, 37532012 PMC10528879

[ref68] Humbert S. Bryson E. A. Cordelières F. P. Connors N. C. Datta S. R. Finkbeiner S. . (2002). The IGF-1/Akt pathway is neuroprotective in Huntington's disease and involves huntingtin phosphorylation by Akt. Dev. Cell 2, 831–837. doi: 10.1016/s1534-5807(02)00188-0, 12062094

[ref69] Institute for Health Metrics and Evaluation Global Burden of Disease (GBD) Results (2024) Available online at: https://vizhub.healthdata.org/gbd-results/ (Accessed April 16, 2026).

[ref70] Jia B. Liu W. Gu J. Wang J. Lv W. Zhang W. . (2019). MiR-7-5p suppresses stemness and enhances temozolomide sensitivity of drug-resistant glioblastoma cells by targeting Yin Yang 1. Exp. Cell Res. 375, 73–81. doi: 10.1016/j.yexcr.2018.12.016, 30586549

[ref71] Junn E. Lee K. W. Jeong B. S. Chan T. W. Im J. Y. Mouradian M. M. (2009). Repression of alpha-synuclein expression and toxicity by microRNA-7. Proc. Natl. Acad. Sci. USA 106, 13052–13057. doi: 10.1073/pnas.0906277106, 19628698 PMC2722353

[ref72] Kacem H. d'Angelo M. Qosja E. Topi S. Castelli V. Cimini A. (2025). The inflammatory bridge between type 2 diabetes and neurodegeneration: a molecular perspective. Int. J. Mol. Sci. 26:7566. doi: 10.3390/ijms26157566 40806709 PMC12347821

[ref73] Kar A. N. Lee S. J. Sahoo P. K. Thames E. Yoo S. Houle J. D. . (2021). MicroRNAs 21 and 199a-3p regulate axon growth potential through modulation of Pten and mTor mRNAs. eNeuro 8:ENEURO.0155-21.2021. doi: 10.1523/ENEURO.0155-21.2021, 34326064 PMC8362682

[ref74] Katsuno M. Tanaka F. Adachi H. Banno H. Suzuki K. Watanabe H. . (2012). Pathogenesis and therapy of spinal and bulbar muscular atrophy (SBMA). Prog. Neurobiol. 99, 246–256. doi: 10.1016/j.pneurobio.2012.05.007, 22609045

[ref75] Kefas B. Godlewski J. Comeau L. Li Y. Abounader R. Hawkinson M. . (2008). microRNA-7 inhibits the epidermal growth factor receptor and the Akt pathway and is down-regulated in glioblastoma. Cancer Res. 68, 3566–3572. doi: 10.1158/0008-5472.CAN-07-6639, 18483236

[ref76] Kellar D. Craft S. (2020). Brain insulin resistance in Alzheimer's disease and related disorders: mechanisms and therapeutic approaches. Lancet Neurol. 19, 758–766. doi: 10.1016/S1474-4422(20)30231-3, 32730766 PMC9661919

[ref77] Khayachi A. Nunes A. Alda M. Rouleau G. A. (2025). The overlooked role of metabolic disorders in bipolar disorder. Neurosci. Biobehav. Rev. 174:106203. doi: 10.1016/j.neubiorev.2025.106203, 40339674

[ref78] Kim A. H. Reimers M. Maher B. Williamson V. McMichael O. McClay J. L. . (2010). MicroRNA expression profiling in the prefrontal cortex of individuals affected with schizophrenia and bipolar disorders. Schizophr. Res. 124, –191. doi: 10.1016/j.schres.2010.07.002, 20675101 PMC4373420

[ref79] Kim Y. Vadodaria K. C. Lenkei Z. Kato T. Gage F. H. Marchetto M. C. . (2019). Mitochondria, metabolism, and redox mechanisms in psychiatric disorders. Antioxid. Redox Signal. 31, 275–317. doi: 10.1089/ars.2018.7606, 30585734 PMC6602118

[ref80] Kolb H. Kempf K. Martin S. (2023). Insulin and aging - a disappointing relationship. Front. Endocrinol. 14:1261298. doi: 10.3389/fendo.2023.1261298, 37854186 PMC10579801

[ref81] Kouli A. Torsney K. M. Kuan W. L. (2018). “Parkinson’s disease: etiology, neuropathology, and pathogenesis,” in Parkinson’s Disease: Pathogenesis and Clinical Aspects, eds. Stoker T. B. Greenland J. C. (Brisbane: Codon Publications). 30702842

[ref82] Kumari S. Dhapola R. Reddy D. H. (2023). Apoptosis in Alzheimer's disease: insight into the signaling pathways and therapeutic avenues. Apoptosis 28, 943–957. doi: 10.1007/s10495-023-01848-y, 37186274

[ref83] Kushwaha S. Lakhanpal V. Elangovan A. Iyer M. Kinoshita M. Narayanasamy A. . (2026). Unravelling the novel insights between miR-7 and Parkinson's disease (PD). Neurol. Sci. 47:310. doi: 10.1007/s10072-026-08834-7, 41762282

[ref84] Kutwin M. Sosnowska-Ławnicka M. Nasiłowska B. Lange A. Wierzbicki M. Jaworski S. (2024). The delivery of mimic miRNA-7 into glioblastoma cells and tumour tissue by graphene oxide Nanosystems. Nanotechnol. Sci. Appl. 17, 167–188. doi: 10.2147/NSA.S469193, 39280996 PMC11402368

[ref85] Lam X. J. Xu B. Yeo P. L. Cheah P. S. Ling K. H. (2023). Mitochondria dysfunction and bipolar disorder: from pathology to therapy. IBRO Neurosci. Rep. 14, 407–418. doi: 10.1016/j.ibneur.2023.04.002, 37388495 PMC10300489

[ref86] LaPierre M. P. Lawler K. Godbersen S. Farooqi I. S. Stoffel M. (2022). MicroRNA-7 regulates melanocortin circuits involved in mammalian energy homeostasis. Nat. Commun. 13:5733. doi: 10.1038/s41467-022-33367-w, 36175420 PMC9522793

[ref87] Lee S. Y. Lu R. B. Wang L. J. Chang C. H. Lu T. Wang T. Y. . (2020). Serum miRNA as a possible biomarker in the diagnosis of bipolar II disorder. Sci. Rep. 10:1131. doi: 10.1038/s41598-020-58195-0, 31980721 PMC6981268

[ref88] Li B. Jiang Y. Xu Y. Li Y. Li B. (2019). Identification of miRNA-7 as a regulator of brain-derived neurotrophic factor/α-synuclein axis in atrazine-induced Parkinson's disease by peripheral blood and brain microRNA profiling. Chemosphere 233, 542–548. doi: 10.1016/j.chemosphere.2019.05.064, 31185338

[ref89] Li Y. Lv Z. Dai Y. Yu L. Zhang L. Wang K. . (2025). The global, regional, and national burden of parkinson's disease in 204 countries and territories, 1990-2021: a systematic analysis for the global burden of disease study 2021. BMC Public Health 25:3047. doi: 10.1186/s12889-025-23492-8, 40940662 PMC12427116

[ref90] Li K. Zhu L. Lv H. Bai Y. Guo C. He K. (2024). The role of microRNA in schizophrenia: a scoping review. Int. J. Mol. Sci. 25:7673. doi: 10.3390/ijms25147673, 39062916 PMC11277492

[ref91] Liberti M. V. Locasale J. W. (2016). The Warburg effect: how does it benefit Cancer cells? Trends Biochem. Sci. 41, 211–218. doi: 10.1016/j.tibs.2015.12.001, 26778478 PMC4783224

[ref92] Liu Y. P. Berkhout B. (2011). miRNA cassettes in viral vectors: problems and solutions. Biochim. Biophys. Acta 1809, 732–745. doi: 10.1016/j.bbagrm.2011.05.014, 21679781

[ref93] Liu X. Dong L. Jiang Z. Song M. Yan P. (2024). Identifying the differentially expressed peripheral blood microRNAs in psychiatric disorders: a systematic review and meta-analysis. Front. Psych. 15:1390366. doi: 10.3389/fpsyt.2024.1390366, 38827444 PMC11140110

[ref94] Liu C. Liu J. Chen H. (2024). Overexpression of miR-7-5p promoted fracture healing through inhibiting LRP4 and activating Wnt/β-catenin pathway. Int J Low Extrem Wounds 23, 86–91. doi: 10.1177/15347346231157443, 36883209

[ref95] Liu Z. Liu Y. Li L. Xu Z. Bi B. Wang Y. . (2014). MiR-7-5p is frequently downregulated in glioblastoma microvasculature and inhibits vascular endothelial cell proliferation by targeting RAF1. Tumour Biol. 35, 10177–10184. doi: 10.1007/s13277-014-2318-x, 25027403

[ref96] Longinetti E. Fang F. (2019). Epidemiology of amyotrophic lateral sclerosis: an update of recent literature. Curr. Opin. Neurol. 32, 771–776. doi: 10.1097/WCO.0000000000000730, 31361627 PMC6735526

[ref97] López-Otín C. Blasco M. A. Partridge L. Serrano M. Kroemer G. (2013). The hallmarks of aging. Cell 153, 1194–1217. doi: 10.1016/j.cell.2013.05.039, 23746838 PMC3836174

[ref98] Louis D. N. Perry A. Reifenberger G. von Deimling A. Figarella-Branger D. Cavenee W. K. . (2016). The 2016 World Health Organization classification of tumors of the central nervous system: a summary. Acta Neuropathol. 131, 803–820. doi: 10.1007/s00401-016-1545-1, 27157931

[ref99] Lundstrom K. (2020). Viral vectors applied for RNAi-based antiviral therapy. Viruses 12:924. doi: 10.3390/v12090924, 32842491 PMC7552024

[ref100] Maltseva S. Kerr D. Turke M. Adams E. J. Lee K. Y. C. (2024). Parkinson's disease-associated mutations in α-synuclein alters its lipid-bound state. Biophys. J. 123, 1610–1619. doi: 10.1016/j.bpj.2024.05.002, 38702883 PMC11213968

[ref101] Mao X. Zhou J. Kong L. Zhu L. Yang D. Zhang Z. (2023). A peptide encoded by lncRNA MIR7-3 host gene (MIR7-3HG) alleviates dexamethasone-induced dysfunction in pancreatic β-cells through the PI3K/AKT signaling pathway. Biochem. Biophys. Res. Commun. 647, 62–71. doi: 10.1016/j.bbrc.2023.01.004, 36731335

[ref102] Martínez-Garcia M. Álvarez-Linera J. Carrato C. Ley L. Luque R. Maldonado X. . (2018). SEOM clinical guidelines for diagnosis and treatment of glioblastoma (2017). Clinic. Transl. Oncol. 20, 22–28. doi: 10.1007/s12094-017-1763-6, 29086250 PMC5785619

[ref103] Martín-Martín Y. Pérez-García A. Torrecilla-Parra M. Fernández-de Frutos M. Pardo-Marqués V. Casarejos M. J. . (2022). New insights on the regulation of the insulin-degrading enzyme: role of microRNAs and RBPs. Cells 11:2538. doi: 10.3390/cells11162538, 36010613 PMC9406717

[ref104] Masrori P. Van Damme P. (2020). Amyotrophic lateral sclerosis: a clinical review. Eur. J. Neurol. 27, 1918–1929. doi: 10.1111/ene.14393, 32526057 PMC7540334

[ref105] Medina A. Mahjoub Y. Shaver L. Pringsheim T. (2022). Prevalence and incidence of Huntington's disease: An updated systematic review and Meta-analysis. Mov. Disord. 37, 2327–2335. doi: 10.1002/mds.29228, 36161673 PMC10086981

[ref106] Memczak S. Jens M. Elefsinioti A. Torti F. Krueger J. Rybak A. . (2013). Circular RNAs are a large class of animal RNAs with regulatory potency. Nature 495, 333–338. doi: 10.1038/nature11928, 23446348

[ref107] Michailidis M. Moraitou D. Tata D. A. Kalinderi K. Papamitsou T. Papaliagkas V. (2022). Alzheimer's disease as type 3 diabetes: common pathophysiological mechanisms between Alzheimer's disease and type 2 diabetes. Int. J. Mol. Sci. 23:2687. doi: 10.3390/ijms23052687, 35269827 PMC8910482

[ref108] Mirzaei Z. Barati T. Ebrahimi A. Derakhshan S. M. Khaniani M. S. (2024). The role of miR-7-5p in cancer: function, prognosis, diagnosis, and therapeutic implications. Mol. Biol. Rep. 52:12. doi: 10.1007/s11033-024-10107-5, 39585455

[ref109] Miyazaki Y. Adachi H. Katsuno M. Minamiyama M. Jiang Y. M. Huang Z. . (2012). Viral delivery of miR-196a ameliorates the SBMA phenotype via the silencing of CELF2. Nat. Med. 18, 1136–1141. doi: 10.1038/nm.2791, 22660636

[ref110] Mizuki Y. Sakamoto S. Okahisa Y. Yada Y. Hashimoto N. Takaki M. . (2021). Mechanisms underlying the comorbidity of schizophrenia and type 2 diabetes mellitus. Int. J. Neuropsychopharmacol. 24, 367–382. doi: 10.1093/ijnp/pyaa097, 33315097 PMC8130204

[ref111] Moon H. E. Paek S. H. (2015). Mitochondrial dysfunction in Parkinson's disease. Exp. Neurobiol. 24, 103–116. doi: 10.5607/en.2015.24.2.103, 26113789 PMC4479806

[ref112] Motti D. Bixby J. L. Lemmon V. P. (2012). MicroRNAs and neuronal development. Semin. Fetal Neonatal Med. 17, 347–352. doi: 10.1016/j.siny.2012.07.008, 22906916 PMC3490020

[ref113] Nelson A. T. Trotti D. (2022). Altered bioenergetics and metabolic homeostasis in amyotrophic lateral sclerosis. Neurotherapeutics 19, 1102–1118. doi: 10.1007/s13311-022-01262-3, 35773551 PMC9587161

[ref114] Nikolova E. Georgiev C. Laleva L. Milev M. Spiriev T. Stoyanov S. . (2022). Diagnostic, grading and prognostic role of a restricted miRNAs signature in primary and metastatic brain tumours. Discussion on their therapeutic perspectives. Mol. Gen. Genomics. 297, 357–371. doi: 10.1007/s00438-021-01851-5, 35064290

[ref115] Ning B. F. Ding J. Liu J. Yin C. Xu W. P. Cong W. M. . (2014). Hepatocyte nuclear factor 4α-nuclear factor-κB feedback circuit modulates liver cancer progression. Hepatology 60, 1607–1619. doi: 10.1002/hep.27177, 24752868

[ref116] Okuda H. Xing F. Pandey P. R. Sharma S. Watabe M. Pai S. K. . (2013). miR-7 suppresses brain metastasis of breast cancer stem-like cells by modulating KLF4. Cancer Res. 73, 1434–1444. doi: 10.1158/0008-5472.CAN-12-2037, 23384942 PMC3576138

[ref117] Olmedo-Saura G. Bernardi E. Bojtos L. Martínez-Horta S. Pagonabarraga J. Kulisevsky J. . (2025). Update on the symptomatic treatment of Huntington's disease: from pathophysiology to clinical practice. Int. J. Mol. Sci. 26:6220. doi: 10.3390/ijms26136220, 40650011 PMC12250415

[ref118] Olmo I. G. Olmo R. P. Gonçalves A. N. A. Pires R. G. W. Marques J. T. Ribeiro F. M. (2021). High-throughput sequencing of BACHD mice reveals upregulation of neuroprotective miRNAs at the pre-symptomatic stage of Huntington's disease. ASN Neuro 13:17590914211009857. doi: 10.1177/17590914211009857, 33906482 PMC8718118

[ref119] Ordóñez-Rubiano E. G. Rincón-Arias N. Espinosa S. Shelton W. J. Salazar A. F. Cómbita A. . (2024). The potential of miRNA-based approaches in glioblastoma: an update in current advances and future perspectives. Curr. Res. Pharmacol. Drug Discov. 7:100193. doi: 10.1016/j.crphar.2024.100193, 39055532 PMC11268206

[ref120] Owen M. J. Sawa A. Mortensen P. B. (2016). Schizophrenia. Lancet 388, 86–97. doi: 10.1016/S0140-6736(15)01121-6, 26777917 PMC4940219

[ref121] Pan C. M. Chan K. H. Chen C. H. Jan C. I. Liu M. C. Lin C. M. . (2020). MicroRNA-7 targets T-box 2 to inhibit epithelial-mesenchymal transition and invasiveness in glioblastoma multiforme. Cancer Lett. 493, 133–142. doi: 10.1016/j.canlet.2020.08.024, 32861705

[ref122] Pan T. Rawal P. Wu Y. Xie W. Jankovic J. Le W. (2009). Rapamycin protects against rotenone-induced apoptosis through autophagy induction. Neuroscience 164, 541–551. doi: 10.1016/j.neuroscience.2009.08.014, 19682553

[ref123] Pan J. Yao Q. Wang Y. Chang S. Li C. Wu Y. . (2024). The role of PI3K signaling pathway in Alzheimer's disease. Front. Aging Neurosci. 16:1459025. doi: 10.3389/fnagi.2024.1459025, 39399315 PMC11466886

[ref124] Pavlova N. N. Zhu J. Thompson C. B. (2022). The hallmarks of cancer metabolism: still emerging. Cell Metab. 34, 355–377. doi: 10.1016/j.cmet.2022.01.007, 35123658 PMC8891094

[ref125] Pei Y. Zhang H. Lu K. Tang X. Li J. Zhang E. . (2022). Circular RNA circRNA_0067934 promotes glioma development by modulating the microRNA miR-7/ Wnt/β-catenin axis. Bioengineered 13, 5792–5802. doi: 10.1080/21655979.2022.2033382, 35213267 PMC8973834

[ref126] Peng Y. Croce C. M. (2016). The role of MicroRNAs in human cancer. Signal Transduct. Target. Ther. 1:15004. doi: 10.1038/sigtrans.2015.4, 29263891 PMC5661652

[ref127] Rademacher K. Nakamura K. (2024). Role of dopamine neuron activity in Parkinson's disease pathophysiology. Exp. Neurol. 373:114645. doi: 10.1016/j.expneurol.2023.114645, 38092187

[ref128] Ratovitski T. Chighladze E. Arbez N. Boronina T. Herbrich S. Cole R. N. . (2012). Huntingtin protein interactions altered by polyglutamine expansion as determined by quantitative proteomic analysis. Cell Cycle 11, 2006–2021. doi: 10.4161/cc.20423, 22580459 PMC3359124

[ref129] Reczek D. Schwake M. Schröder J. Hughes H. Blanz J. Jin X. . (2007). LIMP-2 is a receptor for lysosomal mannose-6-phosphate-independent targeting of beta-glucocerebrosidase. Cell 131, 770–783. doi: 10.1016/j.cell.2007.10.018, 18022370

[ref130] Reddy S. D. Ohshiro K. Rayala S. K. Kumar R. (2008). MicroRNA-7, a homeobox D10 target, inhibits p21-activated kinase 1 and regulates its functions. Cancer Res. 68, 8195–8200. doi: 10.1158/008-5472.CAN-08-2103, 18922890 PMC3636563

[ref131] Roberts T. C. Langer R. Wood M. J. A. (2020). Advances in oligonucleotide drug delivery. Nat. Rev. Drug Discov. 19, 673–694. doi: 10.1038/s41573-020-0075-7, 32782413 PMC7419031

[ref132] Rodríguez-Muela N. (2020). Autophagy in motor neuron diseases. Prog. Mol. Biol. Transl. Sci. 172, 157–202. doi: 10.1016/bs.pmbts.2020.03.009, 32620242

[ref133] Roosterman D. Cottrell G. S. (2021). The two-cell model of glucose metabolism: a hypothesis of schizophrenia. Mol. Psychiatry 26, 1738–1747. doi: 10.1038/s41380-020-00980-4, 33402704 PMC8440173

[ref134] Rostovtseva T. K. Gurnev P. A. Protchenko O. Hoogerheide D. P. Yap T. L. Philpott C. C. . (2015). α-Synuclein shows high affinity interaction with voltage-dependent Anion Channel, suggesting mechanisms of mitochondrial regulation and toxicity in Parkinson disease. J. Biol. Chem. 290, 18467–18477. doi: 10.1074/jbc.M115.641746, 26055708 PMC4513106

[ref135] Rovini A. Gurnev P. A. Beilina A. Queralt-Martín M. Rosencrans W. Cookson M. R. . (2020). Molecular mechanism of olesoxime-mediated neuroprotection through targeting α-synuclein interaction with mitochondrial VDAC. Cell. Mol. Life Sci. 77, 3611–3626. doi: 10.1007/s00018-019-03386-w, 31760463 PMC7244372

[ref136] Rudajev V. Novotny J. (2022). Cholesterol as a key player in amyloid β-mediated toxicity in Alzheimer's disease. Front. Mol. Neurosci. 15:937056. doi: 10.3389/fnmol.2022.937056, 36090253 PMC9453481

[ref137] Rudnik S. Heybrock S. Coyaud E. Xu Z. Neculai D. Raught B. . (2024). The lysosomal lipid transporter LIMP-2 is part of lysosome-ER STARD3-VAPB-dependent contact sites. J. Cell Sci. 137:jcs261810. doi: 10.1242/jcs.261810, 39370902

[ref138] Rusmini P. Polanco M. J. Cristofani R. Cicardi M. E. Meroni M. Galbiati M. . (2015). Aberrant Autophagic response in the muscle of a Knock-in mouse model of spinal and bulbar muscular atrophy. Sci. Rep. 5:15174. doi: 10.1038/srep15174, 26490709 PMC4614888

[ref139] Russman B. S. (2007). Spinal muscular atrophy: clinical classification and disease heterogeneity. J. Child Neurol. 22, 946–951. doi: 10.1177/0883073807305673, 17761648

[ref140] Sabaie H. Mazaheri Moghaddam M. Mazaheri Moghaddam M. Amirinejad N. Asadi M. R. Daneshmandpour Y. . (2021). Long non-coding RNA-associated competing endogenous RNA axes in the olfactory epithelium in schizophrenia: a bioinformatics analysis. Sci. Rep. 11:24497. doi: 10.1038/s41598-021-04326-0, 34969953 PMC8718521

[ref141] Sanek N. A. Young W. S. (2012). Investigating the in vivo expression patterns of miR-7 microRNA family members in the adult mouse brain. MicroRNA 1, 11–18. doi: 10.2174/2211536611201010011, 25048085

[ref142] Sarma M. Chatterjee S. (2024). Etiology of late-onset Alzheimer’s disease, biomarker efficacy, and the role of machine learning in stage diagnosis. Diagnostics 14:2640. doi: 10.3390/diagnostics14232640, 39682548 PMC11640179

[ref143] Saudou F. Humbert S. (2016). The biology of huntingtin. Neuron 89, 910–926. doi: 10.1016/j.neuron.2016.02.003, 26938440

[ref144] Schreurs L. D. Vom Stein A. F. Jünger S. T. Timmer M. Noh K. W. Buettner R. . (2025). The immune landscape in brain metastasis. Neuro-Oncology 27, 50–62. doi: 10.1093/neuonc/noae219, 39403738 PMC11726252

[ref145] Schultz J. L. Neema M. Nopoulos P. C. (2023). Unravelling the role of huntingtin: from neurodevelopment to neurodegeneration. Brain 146, 4408–4410. doi: 10.1093/brain/awad353, 37816304 PMC10629758

[ref146] Seong M. Lee J. Kang H. (2019). Hypoxia-induced regulation of mTOR signaling by miR-7 targeting REDD1. J. Cell. Biochem. 120, 4523–4532. doi: 10.1002/jcb.27740, 30302791

[ref147] Shen D. F. Qi H. P. Ma C. Chang M. X. Zhang W. N. Song R. R. (2021). Astaxanthin suppresses endoplasmic reticulum stress and protects against neuron damage in Parkinson's disease by regulating miR-7/SNCA axis. Neurosci. Res. 165, 51–60. doi: 10.1016/j.neures.2020.04.003, 32333925

[ref148] Shen C. L. Tsai S. J. Lin C. P. Yang A. C. (2023). Progressive brain abnormalities in schizophrenia across different illness periods: a structural and functional MRI study. Schizophrenia 9:2. doi: 10.1038/s41537-022-00328-7, 36604437 PMC9816110

[ref149] Shukla A. Gupta P. Singh R. Mishra D. P. (2018). Glycolytic inhibitor 2-Deoxy-d-glucose activates migration and invasion in glioblastoma cells through modulation of the miR-7-5p/TFF3 signaling pathway. Biochem. Biophys. Res. Commun. 499, 829–835. doi: 10.1016/j.bbrc.2018.04.001, 29621542

[ref150] Sian-Hulsmann J. Riederer P. Michel T. M. (2024). Metabolic dysfunction in Parkinson’s disease: unraveling the glucose–lipid connection. Biomedicine 12:2841. doi: 10.3390/biomedicines12122841, 39767747 PMC11673947

[ref151] Singh A. Agrawal N. (2022). Metabolism in Huntington's disease: a major contributor to pathology. Metab. Brain Dis. 37, 1757–1771. doi: 10.1007/s11011-021-00844-y, 34704220

[ref152] Singh N. Miner A. Hennis L. Mittal S. (2021). Mechanisms of temozolomide resistance in glioblastoma - a comprehensive review. Cancer Drug Resist. 4, 17–43. doi: 10.20517/cdr.2020.79 34337348 PMC8319838

[ref153] Singh B. Swartz H. A. Cuellar-Barboza A. B. Schaffer A. Kato T. Dols A. . (2025). Bipolar disorder. Lancet 406, 963–978. doi: 10.1016/S0140-6736(25)01140-7, 40712624 PMC12340982

[ref154] Sipos D. Raposa B. L. Freihat O. Simon M. Mekis N. Cornacchione P. . (2025). Glioblastoma: clinical presentation, multidisciplinary management, and long-term outcomes. Cancer 17:146. doi: 10.3390/cancers17010146, 39796773 PMC11719842

[ref155] Smigielski L. Jagannath V. Rössler W. Walitza S. Grünblatt E. (2020). Epigenetic mechanisms in schizophrenia and other psychotic disorders: a systematic review of empirical human findings. Mol. Psychiatry 25, 1718–1748. doi: 10.1038/s41380-019-0601-3, 31907379

[ref156] Søkilde R. Kaadt E. Kristensen L. S. Venø M. T. Kjems J. Nyengaard J. R. . (2025). Application of microRNA in situ hybridization on long-term stored human formalin-fixed paraffin-embedded brain samples from psychiatric patients. Mol. Neurobiol. 62, 12736–12746. doi: 10.1007/s12035-025-05077-z, 40450086 PMC12433438

[ref157] Song H. T. Sun X. Y. Zhang L. Zhao L. Guo Z. M. Fan H. M. . (2014). A preliminary analysis of association between the down-regulation of microRNA-181b expression and symptomatology improvement in schizophrenia patients before and after antipsychotic treatment. J. Psychiatr. Res. 54, 134–140. doi: 10.1016/j.jpsychires.2014.03.008, 24694668

[ref158] Sonnay S. Gruetter R. Duarte J. M. N. (2017). How energy metabolism supports cerebral function: insights from ^13^C magnetic resonance studies *in vivo*. Front. Neurosci. 11:288. doi: 10.3389/fnins.2017.00288, 28603480 PMC5445183

[ref159] Soria Lopez J. A. González H. M. Léger G. C. (2019). Alzheimer's disease. Handb. Clinic. Neurol. 167, 231–255. doi: 10.1016/B978-0-12-804766-8.00013-3 31753135

[ref160] Srikantan S. Tominaga K. Gorospe M. (2012). Functional interplay between RNA-binding protein HuR and microRNAs. Curr. Protein Pept. Sci. 13, 372–379. doi: 10.2174/138920312801619394, 22708488 PMC3535178

[ref161] Starhof C. Hejl A. M. Heegaard N. H. H. Carlsen A. L. Burton M. Lilje B. . (2019). The biomarker potential of cell-free microRNA from cerebrospinal fluid in parkinsonian syndromes. Mov. Disord. 34, 246–254. doi: 10.1002/mds.27542, 30557454

[ref162] Su J. Cole J. Fox M. A. (2017). Loss of interneuron-derived collagen XIX leads to a reduction in Perineuronal nets in the mammalian telencephalon. ASN Neuro 9:1759091416689020. doi: 10.1177/1759091416689020, 28090790 PMC5298462

[ref163] Su F. Kim W. S. Halliday G. M. Fu Y. (2026). Alpha-Synuclein in neurodegeneration: from shared biology to disease-specific phenotypes. Cells 15:451. doi: 10.3390/cells15050451, 41827884 PMC12985178

[ref164] Su X. Wang W. Ma S. Ning H. Chen J. (2022). Regulation effect of miR-7 on intervening colorectal cancer rats with HP infection through Akt/GSK-3β/β-catenin pathway. Cell. Mol. Biol. (Noisy-le-Grand) 68, 135–139. doi: 10.14715/cmb/2022.68.6.22, 36227663

[ref165] Sun X. Y. Lu J. Zhang L. Song H. T. Zhao L. Fan H. M. . (2015a). Aberrant microRNA expression in peripheral plasma and mononuclear cells as specific blood-based biomarkers in schizophrenia patients. J. Clinic. Neurosci. 22, 570–574. doi: 10.1016/j.jocn.2014.08.018, 25487174

[ref166] Sun X. Y. Zhang J. Niu W. Guo W. Song H. T. Li H. Y. . (2015b). A preliminary analysis of microRNA as potential clinical biomarker for schizophrenia. Am. J. Med. Genet. Part B Neuropsychiatr. Genet. 168B, 170–178. doi: 10.1002/ajmg.b.32292, 25656957

[ref167] Tetsu O. McCormick F. (1999). Beta-catenin regulates expression of cyclin D1 in colon carcinoma cells. Nature 398, 422–426. doi: 10.1038/18884, 10201372

[ref168] Torrecilla-Parra M. Pardo-Marqués V. Fuentes-Fayos A. C. G-García M. E. Fernández-de Frutos M. López-Aceituno J. L. . (2025). MiR-7 inhibits progression of glioblastoma by impairing autophagy resolution, energy metabolism and ECM remodeling. J. Exp. Clin. Cancer Res. 44:237. doi: 10.1186/s13046-025-03504-6, 40813676 PMC12351809

[ref169] Tracey T. J. Steyn F. J. Wolvetang E. J. Ngo S. T. (2018). Neuronal lipid metabolism: multiple pathways driving functional outcomes in health and disease. Front. Mol. Neurosci. 11:10. doi: 10.3389/fnmol.2018.00010, 29410613 PMC5787076

[ref170] Tsai K. W. Yang Y. F. Wang L. J. Pan C. C. Chang C. H. Chiang Y. C. . (2024). Correlation of potential diagnostic biomarkers (circulating miRNA and protein) of bipolar II disorder. J. Psychiatr. Res. 172, 254–260. doi: 10.1016/j.jpsychires.2024.02.046, 38412788

[ref171] Unzueta-Larrinaga P. Cuesta-Vega E. Barrena-Barbadillo R. Olabarrieta E. Recio-Barbero M. Horrillo I. . (2026). Extracellular matrix dysfunction and synaptic alterations in schizophrenia. Mol. Psychiatry 31, 396–406. doi: 10.1038/s41380-025-03154-2, 40858782 PMC12700823

[ref172] Walker F. O. (2007). Huntington's disease. Lancet 369, 218–228. doi: 10.1016/S0140-6736(07)60111-1, 17240289

[ref173] Wang X. Becker K. Levine N. Zhang M. Lieberman A. P. Moore D. J. . (2019). Pathogenic alpha-synuclein aggregates preferentially bind to mitochondria and affect cellular respiration. Acta Neuropathol. Commun. 7:41. doi: 10.1186/s40478-019-0696-4, 30871620 PMC6419482

[ref174] Wang L. Tian S. Ruan S. Wei J. Wei S. Chen W. . (2024). Neuroprotective effects of cordycepin on MPTP-induced Parkinson's disease mice via suppressing PI3K/AKT/mTOR and MAPK-mediated neuroinflammation. Free Radic. Biol. Med. 216, 60–77. doi: 10.1016/j.freeradbiomed.2024.02.023, 38479634

[ref175] Wang X. Wang J. An Z. Yang A. Qiu M. Tan Z. (2023). CircXPO1 promotes glioblastoma malignancy by sponging miR-7-5p. Cells 12:831. doi: 10.3390/cells12060831, 36980172 PMC10047377

[ref176] Wang Y. Wang Q. Song J. (2017). Inhibition of autophagy potentiates the proliferation inhibition activity of microRNA-7 in human hepatocellular carcinoma cells. Oncol. Lett. 14, 3566–3572. doi: 10.3892/ol.2017.6573, 28927113 PMC5588049

[ref177] Westphal C. H. Chandra S. S. (2013). Monomeric synucleins generate membrane curvature. J. Biol. Chem. 288, 1829–1840. doi: 10.1074/jbc.M112.418871, 23184946 PMC3548493

[ref178] Wilton-Clark H. Al-Aghbari A. Yang J. Yokota T. (2023). Advancing epidemiology and genetic approaches for the treatment of spinal and bulbar muscular atrophy: focus on prevalence in the indigenous population of Western Canada. Genes 14:1634. doi: 10.3390/genes14081634, 37628685 PMC10454234

[ref179] World Health Organization Bipolar disorder: key facts (2024) Available online at: https://www.who.int/news-room/fact-sheets/detail/bipolar-disorder (Accessed April 24, 2026).

[ref180] World Health Organization (2025) Schizophrenia. Available online at: https://www.who.int/news-room/fact-sheets/detail/schizophrenia (Accessed April 23, 2026).

[ref181] Wu D. G. Wang Y. Y. Fan L. G. Luo H. Han B. Sun L. H. . (2011). MicroRNA-7 regulates glioblastoma cell invasion via targeting focal adhesion kinase expression. Chin. Med. J. 124, 2616–2621. 22040413

[ref182] Xu N. Lian Y. J. Dai X. Wang Y. J. (2017). miR-7 increases cisplatin sensitivity of gastric Cancer cells through suppressing mTOR. Technol. Cancer Res. Treat. 16, 1022–1030. doi: 10.1177/1533034617717863, 28693382 PMC5762063

[ref183] Yin C. Y. Kong W. Jiang J. Xu H. Zhao W. (2019). miR-7-5p inhibits cell migration and invasion in glioblastoma through targeting SATB1. Oncol. Lett. 17, 1819–1825. doi: 10.3892/ol.2018.9777, 30675243 PMC6341908

[ref184] Yuan M. Cao Z. Li Q. Liu R. Wang J. Xue W. . (2024). Fasting-induced miR-7a-5p in AgRP neurons regulates food intake. Metab. Clin. Exp. 158:155959. doi: 10.1016/j.metabol.2024.155959, 38942170

[ref185] Zacharjasz J. Sztachera M. Smuszkiewicz M. Piwecka M. (2024). Micromanaging the neuroendocrine system - a review on miR-7 and the other physiologically relevant miRNAs in the hypothalamic-pituitary axis. FEBS Lett. 598, 1557–1575. doi: 10.1002/1873-3468.14948, 38858179

[ref186] Zhang J. Sun X. Y. Zhang L. Y. (2015). MicroRNA-7/Shank3 axis involved in schizophrenia pathogenesis. J. Clinic. Neurosci. 22, 1254–1257. doi: 10.1016/j.jocn.2015.01.031, 25882257

[ref187] Zhang J. Zhao M. Yan R. Liu J. Maddila S. Junn E. . (2021). MicroRNA-7 protects against neurodegeneration induced by α-Synuclein preformed fibrils in the mouse brain. Neurotherapeutics 18, 2529–2540. doi: 10.1007/s13311-021-01130-6, 34697773 PMC8804150

[ref188] Zhao W. Q. De Felice F. G. Fernandez S. Chen H. Lambert M. P. Quon M. J. . (2008). Amyloid beta oligomers induce impairment of neuronal insulin receptors. FASEB J. 22, 246–260. doi: 10.1096/fj.06-7703com, 17720802

[ref189] Zhao X. D. Lu Y. Y. Guo H. Xie H. H. He L. J. Shen G. F. . (2015). MicroRNA-7/NF-κB signaling regulatory feedback circuit regulates gastric carcinogenesis. J. Cell Biol. 210, 613–627. doi: 10.1083/jcb.201501073, 26261179 PMC4539989

[ref190] Zhao H. Xu J. Pang L. Zhang Y. Fan H. Liu L. . (2015). Genome-wide DNA methylome reveals the dysfunction of intronic microRNAs in major psychosis. BMC Med. Genet. 8:62. doi: 10.1186/s12920-015-0139-4, 26462620 PMC4604612

[ref191] Zhou Y. Lu M. Du R. H. Qiao C. Jiang C. Y. Zhang K. Z. . (2016). MicroRNA-7 targets nod-like receptor protein 3 inflammasome to modulate neuroinflammation in the pathogenesis of Parkinson's disease. Mol. Neurodegener. 11:28. doi: 10.1186/s13024-016-0094-3, 27084336 PMC4833896

[ref192] Zhou Z. D. Yi L. X. Wang D. Q. Lim T. M. Tan E. K. (2023). Role of dopamine in the pathophysiology of Parkinson's disease. Transl. Neurodegener. 12:44. doi: 10.1186/s40035-023-00378-6, 37718439 PMC10506345

[ref193] Zhou Q. Yu L. Cook J. R. Qiang L. Sun L. (2023). Deciphering the decline of metabolic elasticity in aging and obesity. Cell Metab. 35, 1661–1671.e6. doi: 10.1016/j.cmet.2023.08.001, 37625407 PMC10528724

